# Novel metallomic profiling and non-carcinogenic risk assessment of botanical ingredients for use in herbal, phytopharmaceutical and dietary products using HR-ICP-SFMS

**DOI:** 10.1038/s41598-022-16873-1

**Published:** 2022-10-20

**Authors:** Ciara-Ruth Kenny, Gavin Ring, Aisling Sheehan, Michael A. P. Mc Auliffe, Brigid Lucey, Ambrose Furey

**Affiliations:** 1 CREATE (Centre for Research in Advanced Therapeutic Engineering) and BioExplore, Department of Biological Sciences, Munster Technological University (MTU), Rossa Avenue, Bishopstown, Co. Cork T12 P928 Ireland; 2 Department of Physical Sciences, Munster Technological University (MTU), Rossa Avenue, Bishopstown, Co. Cork T12 P928 Ireland; 3 Centre for Advanced Photonics and Process Analysis (CAPPA), Munster Technological University (MTU), Rossa Avenue, Bishopstown, Co. Cork T12 P928 Ireland

**Keywords:** Environmental sciences, Environmental impact, Plant sciences, Natural variation in plants, Risk factors

## Abstract

Knowledge of element concentrations in botanical extracts is relevant to assure consumer protection given the increased interest in plant-based ingredients. This study demonstrates successful multi-element investigations in order to address the lack of comprehensive profiling data for botanical extracts, while reporting for the first time the metallomic profile(s) of arnica, bush vetch, sweet cicely, yellow rattle, bogbean, rock-tea and tufted catchfly. Key element compositions were quantified using a validated HR-ICP-SFMS method (µg kg^−1^) and were found highly variable between the different plants: Lithium (18–3964); Beryllium (3–121); Molybdenum (75–4505); Cadmium (5–325); Tin (6–165); Barium (747–4646); Platinum (2–33); Mercury (5–30); Thallium (3–91); Lead (12–4248); Bismuth (2–30); Titanium (131–5827); Vanadium (15–1758); Chromium (100–4534); Cobalt (21–652); Nickel (230–6060) and Copper (1910–6340). Compendial permissible limits were not exceeded. Overall, no evidence of a health risk to consumers could be determined from consumption of the investigated plants at reasonable intake rates. Mathematical risk modelling (EDI, CDI, HQ, HI) estimated levels above safe oral thresholds only for Cd (16%) and Pb (8%) from higher intakes of the respective plant-derived material. Following high consumption of certain plants, 42% of the samples were categorised as potentially unsafe due to cumulative exposure to Cu, Cd, Hg and Pb. PCA suggested a potential influence of post-harvest processing on Cr, Ti and V levels in commercially-acquired plant material compared to wild-collected and farm-grown plants. Moreover, a strong correlation was observed between Pb-Bi, Be-V, Bi-Sn, and Tl-Mo occurrence. This study may support future research by providing both robust methodology and accompanying reference profile(s) suitable for the quality evaluation of essential elements and/or metal contaminants in botanical ingredients.

## Introduction

Plants serve a dual role in medicine and food. With reference to regulatory legislation, plant materials used as ingredients in dietary supplements are increasingly described as “botanicals”, whereas plants used as active ingredients in medicinal herbal products are more commonly referred to as “herbs”, regardless, the shared denominator in all such products, are the plants^1^. Essentially, plants are wildcards which are not universally governed by a singular regulatory (EU) framework. In the absence of a harmonised process at European level, their intended use determines their route of regulation, not their phytochemical composition or toxicological properties, as one might expect. The diverse range of products in which they can be used as ingredients, are distinguishable primarily by labelling and health benefits claimed by the manufacturer. Plants and products thereof can be regulated, depending on the proposed use and recommended intake, in accordance with various legislative categories—be it food (general-, novel-, fortified- or genetically modified-food), pharmaceutical, herbal (i.e. Herbal Directive) or cosmetics.

As it currently stands, in the absence of clarity, the same product can be marketed as a foodstuff in one country and a medicinal product in another^2^. This is further complicated with the application of the “*principle of mutual recognition*”, whereby any legally marketed product in one European Member State can be sold in other Member States^3^. The European Commission (E.C.) insists that it is not feasible to pursue harmonisation of botanicals and conditions of usage until further scientific data is available^4^. Furthermore, the application of approved health claims regulated under (E.C.) 1924/2006 to botanical-containing products has resulted in a legal moratorium^4^, largely related to conflicting opinions on the level of scientific rigor required to substantiate such claims^5^. Currently, the BELFRIT [Belgium, France, Italy] list is the only existing amalgamation of accepted “safe” botanicals for use in supplements^1^ and while it serves as a good starting point towards harmonisation, its use is not legally enforceable in Member States. In Ireland, the Food Safety Authority of Ireland (FSAI) has rendered the BELFRIT list(s) as unsuitable for adoption in the regulatory risk management of botanicals on the Irish market. This decision was based on “non-transparency” in the methodologies used^5^, however the FSAI agree that the use of the BELFRIT list(s) in conjunction with the available European Food Safety Authority (EFSA) guidance documentation and Compendium of Botanicals (CoB) are useful preliminary resources for the risk assessment and management of botanical ingredients^5^. The EFSA^6^ acknowledges the market volume expansion for plant-based products and the subsequent need for improved characterisation of an increasing botanical product portfolio and overall harmonisation of the risk assessment process. A recurring opinion among governing bodies is the lack of supporting data in the realm of botanical sciences.

We previously reviewed the adverse human health effects and regulation of metal contaminants in plant-derived food and phytopharmaceuticals^2^. In brief, existing regulatory gaps include a lack of^4,5,7^:

- Nutrient and elemental profiles for high-value plants (e.g. medicinal; aromatic)
- Specification data including permissible or maximum limits for a greater suite of metal contaminants/impurities
- Prospective population intake/consumption data
- Evidence and list of permitted plant species regarded as safe for oral consumption
- Evidence and list of restricted plant species regarded as unsafe for oral consumption
- Assessed health claims for medicinal plants
- Toxicological risk assessments for medicinal plants regarding phytochemical composition
- Advisory labelling statements
- Global monitoring system(s) and pharmacovigilance

In summary, concerns with regards to metal contaminants in botanical ingredients or herbal substances (i.e. starting or raw material) include the unregulated cultivation of medicinal plants, non-enforceable Good Agriculture and Collection Practices (GACP) for raw material of plant origin, and the absence of general specifications and acceptance criteria^2^. Maximum permissible limits or maximum levels (MLs) for elemental impurities in medicinal plants can vary, in some cases substantially, between countries and organisations^2^, such as: Pb (3.0–30.0 mg kg^−1^); As (0.6–5.0 mg kg^−1^); Cd (0.2–4.0 mg kg^−1^); Hg (0.02–1.0 mg kg^−1^) and Cu (10.0–150.0 mg kg^−1^)^8^. Currently, there are no universal limits for inorganic metal impurities in medicinal plants or products thereof—and uniformity may never be achieved. Two plausible solutions to consider are the application of the ICH Q3D guidelines to phytopharmaceuticals, or alternatively, the establishment of an extended suite of toxicologically significant specifications for the control of inorganic metal contaminants, for both MLs and assay procedures in plant-derived products^2^. Exceedance of such limits would not automatically affirm the presence of risk, but more so act as a “trigger” that warrants further investigation. Some authors claim that in processes whereby herbal substances (raw plant material) are found in exceedance of threshold limits for elemental impurities, justification should be waived provided compliance is assured in the final [consumer-ready] product^9^. Others assume the probability of exceeding As, Co, Ni and V limits in herbal drugs is low (using ICH Q3D limits as guidance) and thus general limits for the aforementioned in future editions of the European Pharmacopeia (Ph. Eur.) is not a necessity^10^.

The aim of this current study is to address the lack of comprehensive metallomic (and nutrient) profiling data for economically valuable plant species with medicinal, culinary, agricultural and cosmetic uses. It is the first application of a validated high-resolution inductively coupled plasma sector field mass spectrometry (HR-ICP-SFMS) method to quantitively analyze multiple health-related elements in a variety of common botanicals. The plants species (*n* = 30) analysed are listed below, with the botanical common name in parentheses: *Crataegus laevigata* (**hawthorn**), *Taraxacum officinale* (**dandelion**), *Arnica montana* (**arnica**), *Sambucus nigra* L. (**elder**), *Sambucus nigra* fruct. (**elderberry**), *Sambucus nigra* flos. (**elderflower**), *Calendula officinalis* (**marigold**), *Aesculus hippocastanum* (**horse chestnut**), *Urtica dioica* (**nettle**), *Achillea millefolium* (**yarrow**), *Symphytum officinale* (**comfrey**), *Borago officinalis* (**borage**), *Tussilago farfara* (**coltsfoot**), *Vicia sepium* (**bush vetch**), *Lotus corniculatus* (**birds-foot trefoil**), *Leucanthemum vulgare* (**ox-eye daisy**), *Myrrhis odorata* (**sweet cicely**), *Rhinanthus minor* (**yellow rattle**), *Menyanthes trifoliata* (**bogbean**), *Artemisia vulgaris* (**mugwort**), *Verbascum thapsus* (**great mullein**), *Jasonia glutinosa* L. (DC) (**rock tea**), *Silene saxifraga* L. (**tufted catchfly**), *Salvia officinalis* L. (**Sage**), *Glycyrrhiza glabra* (**liquorice**), *Althaea officinalis* (**marshmallow**), *Lavandula angustifolia* (**lavender**), *Hypericum perforatum* (**St. John’s Wort**), *Melissa officinalis* (**lemon balm**), *Santolina chamaecyparissus* (**cotton lavender**), *Mentha* × *piperita* (**peppermint**) and *Peumus boldus* Molina (**boldo**). These are the first reported metallomic profile(s) to-date for arnica, bush vetch, sweet cicely, yellow rattle, bogbean, rock-tea and tufted catchfly. Each plant species is later discussed individually and comprehensively compared to existing literature.

Another major data gap hindering the risk evaluation of botanical ingredients and products, is the lack of intake and consumption data at European level and the availability of guidance documents. Such critical information is required to facilitate harmonised deterministic and probabilistic risk assessment criteria with the outlook of ensuring botanical safety. We later discuss current limitation and the use of generic input parameters for risk assessing plant matrices, including metal transfer rates, exposure durations and frequency data. For this study, preliminary mathematical risk modelling (EDI, CDI, HQ, HI) was used to estimate the potential non-carcinogenic risk to human health via oral consumption using ‘conservative’ and ‘realistic’ theoretical exposure scenarios. Element profiles were subsequently investigated using principle component analysis (PCA) to examine novel trends or patterns in the data. In addition to providing a blueprint method for future investigations within this field, this work can be utilised as a detailed resource for future metallomic profiling of botanical ingredients or products, as well as quality (e.g. authentication, adulteration or nutritional studies) and environmental phytoremediation studies.

## Methods

### Reagents and materials

Ultrapure milli-Q water (15.0 MΩ cm); trace-metal grade nitric acid (HNO_3_) (*PlasmaPure*, 67–69% *w/w* and SCP Science); *Tune-Up* solution (Thermo Scientific, USA; 1 µg L^−1^). Multi-elemental standard solutions including lithium (Li), beryllium (Be), molybdenum (Mo), cadmium (Cd), tin (Sn), barium (Ba), platinum (Pt), gold (Au), mercury (Hg), thallium (Tl), lead (Pb), bismuth (Bi), magnesium (Mg), aluminium (Al), titanium (Ti), vanadium (V), chromium (Cr), manganese (Mn), iron (Fe), cobalt (Co), nickel (Ni) and copper (Cu) (SCP Science via QMX Laboratories, UK) were used in this study. The internal standards (ISTDs) used in this study were gallium (^71^Ga), scandium (^45^Sc), rhodium (^103^Rh), iridium (^193^Ir) and again these were certified standards traceable to NIST reference materials, sourced from SCP Science. Polymethylpentene (PMP) beakers, volumetric flasks, graduated cylinders, and pipettes were sourced from VWR International Ltd. (Blanchardstown, Dublin 15, Ireland).

#### Instrumentation

The MARS-6™ microwave-accelerated reaction system was used for sample digestion (CEM Corporation, USA). The Thermo Scientific ELEMENT2™ (HR) ICP-SFMS (Thermo Fischer Scientific, Bremen, Germany) was coupled with an ESI autosampler and was used for multi-elemental analysis of samples.

#### Sources of plant material

Botanical samples were sourced in raw bulk format (dried, and: cut, fragmented, powdered, or whole plant/organ) from registered commercial suppliers, wild collections, and cultivated sources (e.g. botanical gardens/farms, herbalists) (see Table 1). These samples at large represent raw starting materials or herbal/botanical ingredients and are not considered consumer-ready products.

**Table 1:** List of botanical samples (*n* = 30 species) analysed in this study.

Sample I.D.	Latin name	Common name	Taxonomic order^a^	Plant part; specification^a^	Origin/source	Type^c^
BT_01	*Crataegus laevigata*	Hawthorn	**Ro**sales	Flower & leaf (**F&L**); cut	China	Commercial (**CM**)
BT_02	*Crataegus laevigata*	Hawthorn	**Ro**sales	Flower & leaf (**F&L**); cut	Eastern Europe	Commercial (**CM**)
BT_03	*Crataegus laevigata*	Hawthorn	**Ro**sales	Flower & leaf (**F&L**); whole	Cork, Ireland	Cultivated (**CL**)
BT_04	*Crataegus laevigata*	Hawthorn	**Ro**sales	Flower & leaf (**F&L**); whole	Cork, Ireland	Wild (**W**)
BT_05	*Crataegus laevigata*	Hawthorn	**Ro**sales	Flower & leaf (**F&L**); whole	Cork, Ireland	Wild (**W**)
BT_06	*Crataegus laevigata*^b^	Hawthorn	**Ro**sales	Fruit (**FR**); whole	Albania	Commercial (**CM**)
BT_07	*Crataegus laevigata*	Hawthorn	**Ro**sales	Fruit (**FR**); whole	Cork, Ireland	Wild (**W**)
BT_08	*Arnica montana*	Arnica	**As**terales	Flower (**FL**); whole	UK	Commercial (**CM**)
BT_09	*Taraxacum officinalis*^b^	Dandelion	**As**terales	Root (**R**); cut	France	Commercial (**CM**)
BT_10	*Taraxacum officinalis*^b^	Dandelion	**As**terales	Leaf (**L**); cut	Netherlands	Commercial (**CM**)
BT_11	*Sambucus nigra* (flos)	Elderflower (Flower)	**Di**psacales	Flower (**FL**); whole	Cork, Ireland	Wild (**W**)
BT_12	*Sambucus nigra* (flos)	Elderflower (Flower)	**Di**psacales	Flower (**FL**); whole, rubbed	Bulgaria	Commercial (**CM**)
BT_13	*Sambucus nigra* (fruct.)	Elderberry (Fruit)	**Di**psacales	Fruit (**FR**); whole	Czech Republic	Commercial (**CM**)
BT_14	*Sambucus nigra* (fruct.)	Elderberry (Fruit)	**Di**psacales	Fruit (**FR**); whole	Cork, Ireland	Wild (**W**)
BT_15	*Sambucus nigra* (fruct.)	Elderberry (Fruit)	**Di**psacales	Fruit (**FR**); whole	Cork, Ireland	Wild (**W**)
BT_16	*Sambucus nigra* (fruct.)	Elderberry (Fruit)	**Di**psacales	Fruit (**FR**); whole	Eastern Europe	Commercial (**CM**)
BT_17	*Calendula officinalis*	Marigold	**As**terales	Flowers (**FL**); whole	U.K	Commercial (**CM**)
BT_18	*Aesculus hippocastanum*	Horse Chestnut	**Sa**pindales	Seed (**SD**); cut	Europe	Commercial (**CM**)
BT_19	*Urtica dioica*^b^	Stinging Nettle	**Ro**sales	Leaves (**L**); cut	Europe	Commercial (**CM**)
BT_20	*Urtica dioica*	Stinging Nettle	**Ro**sales	Root (**R**); cut	UK	Commercial (**CM**)
BT_21	*Achillea millefolium*	Yarrow	**As**terales	Flowers (**FL**); cut	Bulgaria	Commercial (**CM**)
BT_22	*Symphytum officinale*	Comfrey	**Bo**raginales	Root (**R**); cut	Bulgaria	Commercial (**CM**)
BT_23	*Symphytum officinale*	Comfrey	**Bo**raginales	Leaves (**L**); powder	Hungary	Commercial (**CM**)
BT_24	*Symphytum officinale*	Comfrey	**Bo**raginales	Leaves (**L**); cut	Cork, Ireland	Cultivated (**CL**)
BT_25	*Symphytum officinale*	Comfrey	**Bo**raginales	Stem (**ST**); cut	Cork, Ireland	Cultivated (**CL**)
BT_26	*Borago officinalis*	Borage	**Bo**raginales	Aerial (**A**); powder	Germany	Commercial (**CM**)
BT_27	*Borago officinalis*	Borage	**Bo**raginales	Aerial (**A**); cut	Germany	Commercial (**CM**)
BT_28	*Tussilago farfara*	Coltsfoot	**As**terales	Flowers (**FL**); cut	Albania	Commercial (**CM**)
BT_29	*Tussilago farfara*	Coltsfoot	**As**terales	Leaves (**L**); cut	Poland	Commercial (**CM**)
BT_30	*Vicia sepium*	Bush Vetch	**Fa**bales	Aerial (**A**); cut	Ireland	Wild (**W**)
BT_31	*Lotus corniculatus*	Birds-Foot Trefoil	**Fa**bales	Aerial (**A**); cut	Ireland	Wild (**W**)
BT_32	*Leucanthemum vulgare*	Ox-Eye Daisy	**As**terales	Flowers (**FL**); whole	Ireland	Wild (**W**)
BT_33	*Leucanthemum vulgare*	Ox-Eye Daisy	**As**terales	Leaves (**L**); whole	Ireland	Wild (**W**)
BT_34	*Myrrhis odorata*	Sweet Cicely	**Ap**iales	Aerial (**A**); cut	Ireland	Wild (**W**)
BT_35	*Rhinanthus minor*	Yellow Rattle	**La**miales	Aerial (**A**); cut	Ireland	Wild (**W**)
BT_36	*Menyanthes trifoliata*	Bogbean	**As**terales	Aerial (**A**); cut	Ireland	Wild (**W**)
BT_37	*Artemisia vulgaris*	Mugwort	**As**terales	Flower & leaf (**F&L**); cut	Ireland	Wild (**W**)
BT_38	*Verbascum thapsus*	Great Mullein	**La**miales	Leaves (**L**); whole	Sligo	Wild (**W**)
BT_39	*Jasonia glutinosa*	Rock Tea	**As**terales	Aerial (**A**); whole	Spain	Cultivated (**CL**)
BT_40	*Silene saxifraga* L.	Tufted Catchfly	**Ca**ryophyllales	Leaf & stem (**L&ST**); whole	Spain	Cultivated (**CL**)
BT_41	*Salvia officinalis* L.	Sage	**La**miales	Aerial (**A**); whole	Spain	Cultivated (**CL**)
BT_42	*Glycyrrhiza glabra*	Liquorice	**Fa**bales	Root (**R**); cut	Spain	Cultivated (**CL**)
BT_43	*Althaea officinalis*	Marshmallow	**Malv**ales	Root (**R**); cut	Spain	Cultivated (**CL**)
BT_44	*Lavandula angustifolia*	Lavender	**La**miales	Flowers (**FL**); whole	Spain	Cultivated (**CL**)
BT_45	*Hypericum perforatum*	St. John’s Wort	**Ma**lpighiales	Aerial (**A**); whole	Spain	Cultivated (**CL**)
BT_46	*Melissa officinalis*	Lemon Balm	**La**miales	Aerial (**A**); whole	Spain	Cultivated (**CL**)
BT_47	*Santolina chamaecyparissus*	Cotton Lavender	**As**terales	Flowers (**FL**); whole	Spain	Cultivated (**CL**)
BT_48	*Sambucus nigra* L.	Elder	**Di**psacales	Aerial (**A**); whole	Spain	Cultivated (**CL**)
BT_49	*Mentha* × *piperita*	Peppermint	**La**miales	Aerial (**A**); whole	Spain	Cultivated (**CL**)
BT_50	*Peumus boldus* Molina	Boldo	**Lau**rales	Leaves (**L**); cut	Spain	Cultivated (**CL**)

The selected plants analysed in this study are currently under investigation in the Department of Biological Sciences at MTU for various medicinal, culinary and agricultural functionalities.

^a^Taxonomic order, plant part(s) and type(s) categories are underlined in bold for use as labels in PCA figures (“Principle component analysis (PCA)” section). ^b^Specification refers to the level of processing of the final product.

^c^Certified organic plant material.

#### Sample preparation

All samples were acquired in dried format. The dried samples were ground to a fine powder, sieved, and stored in airtight sterile plastic containers at room temperature until required for analysis.

### Microwave-assisted acid digestion

#### Vessel preparation

All experimental MARS Xpress vessels were rinsed in triplicate with deionised water before undergoing the Mars6 Xpress cleaning cycle. Ten millilitres of 5% HNO_3_ (*w/w*) was added to each vessel before initiating the pre-programmed OneTouch “*Express Clean*” programme (*Stages*: 1; *Power*: 100–1800; *Ramp Time*: 15 min; *Hold Time*: 10 min; *Temp*.: 150 °C; *Temp. Guard*: Off) (CEM, 2020). On completion of the cleaning cycle, vessels were again rinsed in triplicate with clean deionised water before being allowed to air dry.

#### Pre-digestion of botanical samples

Samples (0.5 g; dry weight) were accurately weighed and transferred into a pre-cleaned MARS Xpress digestion vessel (*Material*: TFM; *maximum vessel volume*: 55 mL; *operation pressure and temperature*: medium). For the pre-digestion step, concentrated trace-metal grade HNO_3_ (*w/w*; 67–69%; 10 mL) was added to the vessel and gently swirled before securing the inner lid and allowed to stand for 15 min at room temperature. Any gas produced during the pre-digestion step was manually released before securing the vessel and placing it into the MARS-6 carousel.

#### Programmed digestion of botanical (BT) samples

The pre-programmed CEM OneTouch “Plant Material” method was selected (*Stage*: 01; *Power*: 1030–1800 W; *Ramp time*: 20–25: 00 mm/ss; *Hold time*: 10:00 mm/ss; *Pressure*: 800 psi; *Temperature*: 200 °C; *Temp. Guard*: off; *Stirring*: off) (CEM, 2020). After cooling, the digestates were transferred to sterile 15 mL PMP sample tubes. The tubes were gently inverted and vented multiple times to release gaseous build-up before storage at − 24 °C. The above steps were repeated for all botanical samples (BT-01 to BT-50).

### Validated HR-ICP-SFMS multi-elemental analysis (metallomic profiling)

Method performed as per Ring et al.^11^; previously validated in our laboratory (Mass Spectrometry group, Department of Physical Sciences, MTU). Standard/control preparation, instrumental analysis and QC measures are outlined briefly below.

#### Multi-elemental standard and control preparation

Calibration standards (in the range of 0.001–50 µg L^−1^) and controls were prepared as described earlier^11^. To prepare the matrix-spiked controls, a sample was diluted 1:10,000 using 2% (*w/w*) HNO_3_ before being spiked with standard and ISTD stock solutions. The final concentrations of these controls were as follows: A (0.2 µg L^−1^), B (1 µg L^−1^), C (5 µg L^−1^), D (15 µg L^−1^) and E (40 µg L^−1^).

#### Instrumentational analysis

A volume of the initial digested sample (5.0 mL) was diluted to 25 mL in 2% (*w/w*) HNO_3_ and spiked with the internal standard (to a final ISTD concentration of 2.5 µg L^−1^). The samples were placed on the ESI autosampler rack, and analysis of all samples by (HR) ICP-SFMS was performed as per the procedure described earlier^11^.

Heavy metals and trace elements present in the digested botanical samples were analysed using a high-resolution (HR) inductively coupled plasma sector-field mass spectrometer (ICP-SFMS); Thermo Scientific™ Element 2™ High-Resolution (HR) ICP-SFMS. Certified calibration standards (traceable to NIST reference materials), controls (calibration verification standards) and blanks were run prior to sample injections. The diluted sample results determined at the instrument were expressed in parts per billion (ppb = µg L^−1^) and the final concentrations were obtained by calculating back to the original solid sample that was initially weighed out (µg kg^−1^). The following element isotopes were quantified in this study as previously described^11^: ^7^Li, ^9^Be, ^95^Mo, ^111^Cd, ^118^Sn, ^137^Ba, ^195^Pt, ^197^Au, ^202^Hg, ^205^Tl, ^208^Pb, ^209^Bi, ^24^Mg, ^27^Al, ^47^Ti, ^51^V, ^52^Cr, ^55^Mn, ^56^Fe, ^59^Co, ^60^Ni and ^63^Cu. Matrix-spiked controls were analysed at five levels spanning the calibration range (0.2, 1, 5, 15 and 40 µg L^−1^) after every 20 samples. Calibration readback QCs (made up in 2% HNO_3_) were also ran at the end of the analytical sequence to verify the calibration line and instrument performance.

#### Calibration and quality assurance

In Table 2, a summary of the calibrations for each element of interest is presented, as well as the limit of detection/limit of quantification (LOD/LOQ) for each analyte. All elements analysed achieved acceptable linearity (R^2^ ≥ 0.995) across their respective working ranges. These calibrations were used to interpolate the concentrations of samples and matrix-spiked controls.

**Table 2:** Summary of the calibration and quality assurance for each element.

Analyte isotope	ISTD Isotope	Equation of the line	Linear range (µg L^−1^)	No. of calibration points	Correlation coefficient (R^2^)	LOD (ng L^−1^)	LOQ (ng L^−1^)
^7^Li	^71^Ga	y = 661.3x + 17.879	0.001–35	11	R^2^ = 0.9999	< 1.00	1.00
^9^Be	^103^Rh	y = 50.303x − 1.1758	0.001–50	10	R^2^ = 0.9999	0.36	1.18
^95^Mo	^103^Rh	y = 125.66x − 3.494	0.005–50	10	R^2^ = 0.9999	1.64	5.40
^111^Cd	^193^Ir	y = 0.0393x − 0.0011	0.001–50	12	R^2^ = 0.9999	0.28	0.94
^118^Sn	^103^Rh	y = 218.16x – 5.0398	0.005–50	14	R^2^ = 0.9999	1.64	5.42
^137^Ba	^103^Rh	y = 136.42x + 2.7994	0.010–50	11	R^2^ = 0.9999	1.96	6.46
^195^Pt	^193^Ir	y = 0.1414x + 0.0016	0.005–25	11	R^2^ = 0.9999	0.43	1.42
^197^Au	^193^Ir	y = 385.37x − 27.221	0.025–50	8	R^2^ = 0.9999	11.52	25.00
^202^Hg	^193^Ir	y = 102.4x − 2.6221	0.010–35	11	R^2^ = 0.9997	11.79	38.91
^205^Tl	^71^Ga	y = 1.19x − 0.0274	0.001–35	11	R^2^ = 0.9999	0.02	0.07
^208^Pb	^103^Rh	y = 613.49x + 30.858	0.005–50	8	R^2^ = 0.9999	2.52	5.00
^209^Bi	^103^Rh	y = 942.11x − 16.505	0.001–50	12	R^2^ = 0.9999	0.39	1.30
^24^Mg	^45^Sc	y = 0.2058x + 0.3627	0.250–50	7	R^2^ = 0.9993	25.00	100.00
^27^Al	^45^Sc	y = 0.285x + 0.6584	0.025–50	7	R^2^ = 0.9998	10.00	25.00
^47^Ti	^45^Sc	y = 0.0262x + 0.0015	0.100–50	7	R^2^ = 0.9999	14.49	47.83
^51^V	^45^Sc	y = 0.2958x − 0.007	0.010–50	14	R^2^ = 0.9999	1.91	6.29
^52^Cr	^45^Sc	y = 0.2815x + 0.0353	0.025–50	11	R^2^ = 0.9999	10.23	33.76
^55^Mn	^45^Sc	y = 0.3524x + 0.0535	0.010–50	12	R^2^ = 0.9999	4.18	13.79
^56^Fe	^45^Sc	y = 0.2916x + 0.5195	0.250–50	8	R^2^ = 0.9997	25.00	100.00
^59^Co	^45^Sc	y = 0.2896x − 0.0018	0.025–45	13	R^2^ = 0.9997	7.13	23.54
^60^Ni	^45^Sc	y = 0.0686x + 0.0432	0.250–50	8	R^2^ = 0.9998	10.00	189.82
^63^Cu	^45^Sc	y = 0.1351x + 0.0259	0.250–50	8	R^2^ = 0.9996	10.00	250.00

The validity of results was assured through the analysis of matrix-spiked controls at five concentration levels spanning the entire calibration range of the method (0.2 µg L^−1^, 1 µg L^−1^, 5 µg L^−1^, 15 µg L^−1^ and 40 µg L^−1^). Percent recovery (% recovery) was used as the parameter to evaluate calibration/instrument performance, with an acceptance tolerance of 100 ± 25% recovery (i.e. 75–125% of the assigned value for each control concentration). As can be seen in Table 3, all elements achieved acceptable recoveries across each concentration level examined. The acceptable performance of the controls indicated that the calibration was fit for purpose and could be used to accurately determine element concentrations in the samples (BT-1 to BT-50).

**Table 3:** Average % recoveries for matrix-spiked controls analysed by ICP-SFMS (*n* = 5).

Element(s)	Average % recovery (*n* = 5)				
	0.2 µg L^−1^	1 µg L^−1^	5 µg L^−1^	15 µg L^−1^	40 µg L^−1^
Lithium (^7^Li)	116.5	105.3	110.4	107.8	105.8
Beryllium (^9^Be)	116.6	103.6	106.0	107.0	108.6
Molybdenum (^95^Mo)	113.0	89.3	88.9	91.8	97.1
Cadmium (^111^Cd)	114.2	96.4	101.1	100.4	97.4
Tin (^118^Sn)	105.7	95.2	94.2	93.7	94.9
Barium (137Ba)	109.2	92.4	88.9	90.6	93.0
Platinum (^195^Pt)	106.7	98.3	101.7	96.9	95.7
Mercury (^202^Hg)	121.0	99.1	103.6	96.9	92.5
Thallium (^205^Tl)	94.5	85.1	80.4	79.9	80.8
Lead (^208^Pb)	77.8	90.3	87.2	85.0	87.9
Bismuth (^209^Bi)	89.8	90.9	87.4	85.1	87.6
Titanium (^47^Ti)	108.5	92.2	92.9	89.3	89.9
Vanadium (^51^V)	109.1	94.6	90.7	90.2	89.5
Chromium (^52^Cr)	96.0	90.4	89.9	89.4	90.9
Cobalt (^59^Co)	104.2	96.4	91.0	89.7	92.8
Nickel (^60^Ni)^a^	–	100.5	93.1	89.1	91.6
Copper (^63^Cu)^a^	–	106.7	92.9	90.8	92.2

^a^Nickel and copper have LOQs of 0.25 µg L^−1^, which is above the lowest (0.2 µg L^−1^) control level, hence they have not been assessed at the 0.2 µg L^−1^ level.

The concentrations of elements in each sample were determined by ICP-SFMS and a summary of the results (expressed in µg kg^−1^) can be found in Table 5. In some samples, element concentrations were found to be outside the instrument calibration range (Mo, Ba, Tl, Pb, Ti and Cu), and as such, are reported as ‘NR’. In the cases of Au, Al, Fe, Mg and Mn, all samples analysed yielded concentrations that lay outside the calibration range and, therefore, these elements have been removed from the table entirely. Where sample concentrations were below the LOQ, final concentrations have been reported as < LOQ.

### Principle component analysis (PCA)

In this study, a correlation matrix (see Supplementary Table S1 online) was used to determine the relationships among the studied elements.

**Figure 1:**
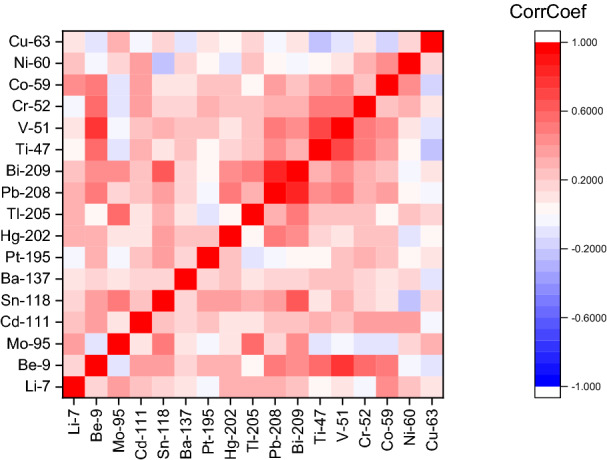
2-D cross-correlation plot.

The 2-D cross-correlation plot (Fig. 1) depicts the elements that tend to occur together. The closer to 1.0, the higher the correlation. Strong correlations (i.e. red gradient) above 0.6 were observed amongst element isotopes such as:

- ^208^Pb and ^209^Bi
- ^9^Be and ^51^V
- ^209^Bi and ^118^Sn
- ^205^Tl and ^95^Mo.

Conversely, negatively correlated elements are represented in blue, such as ^118^Sn and ^60^Ni.

Principal component analysis (PCA) was performed in the Unscrambler™ v11 and plotted using Origin Plotting Software. The concentrations of elements were used as discriminating variables. Eight categories were available including: **source** (*geographical origin*), **type** (i.e. *commercial, cultivated or wild*), **taxonomic order** (e.g., ***Ap****iales, ****As****terales, ****Bo****raginales, ****Ca****ryophyllales, ****Di****psacales, ****Fa****bales, ****La****miales, ****Lau****rales, ****Ma****lpighiales, ****Malv****ales, ****Ro****sales and ****Sa****pindales*), **common name**, **plant part** (above (e.g. *seed, flower, leaf, stem or combinations thereof*) and below ground (e.g. *root*)), and **final-product specification** (e.g. *powdered, cut or whole organ*).

**Figure 2:**
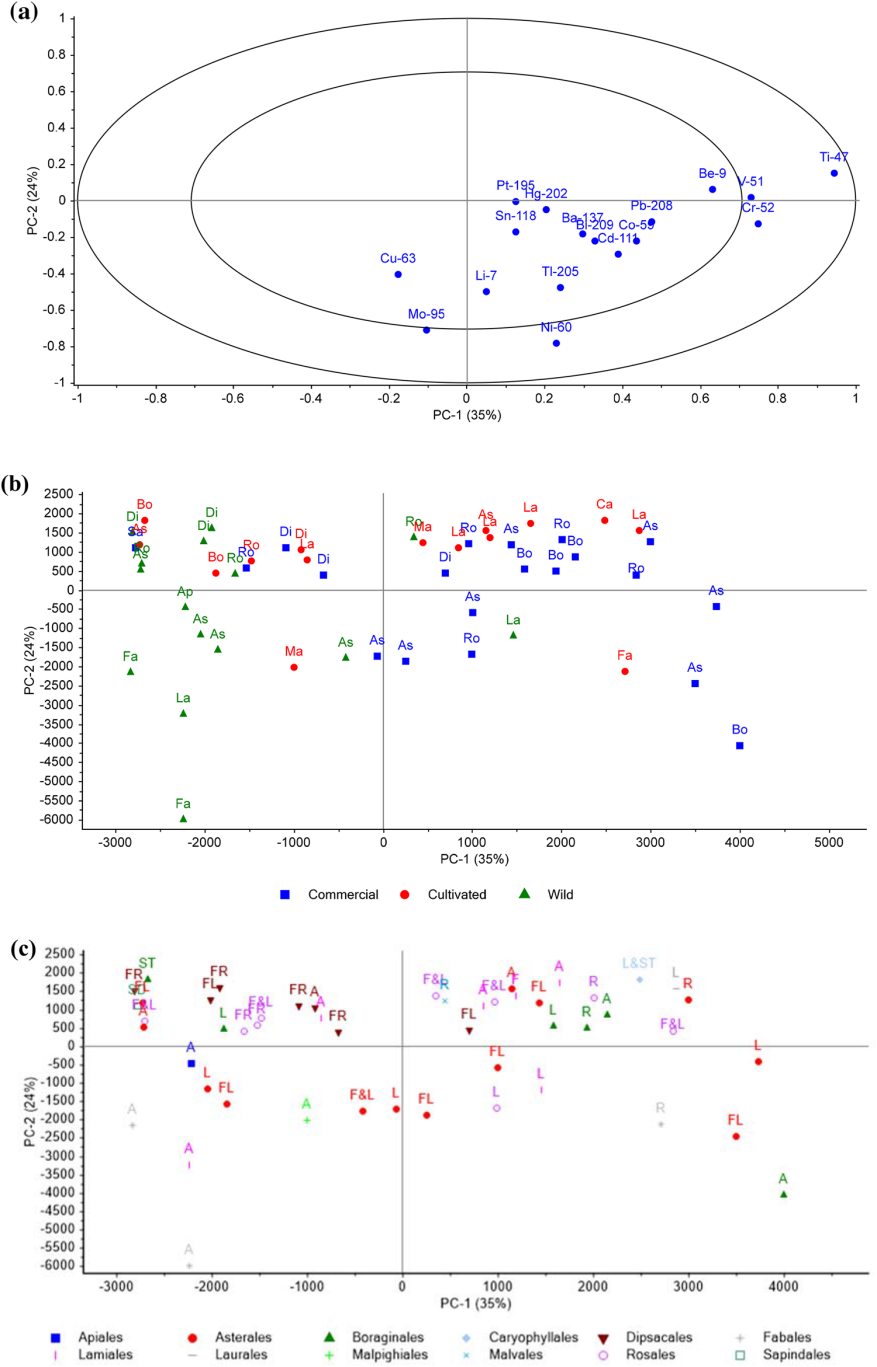
PCA (PC1vPC2) correlation loadings (**a**) and score plots grouped by (**b**) type and labelled by taxonomic order, and (**c**) grouped by taxonomic order and labelled by plant part. Taxonomic orders (e.g. Ro = Rosales) and plant part (e.g. F&L = Flower and Leaf; R = Root; L&ST = Leaf and Stem) abbreviated as per Table 1.

**Figure 3:**
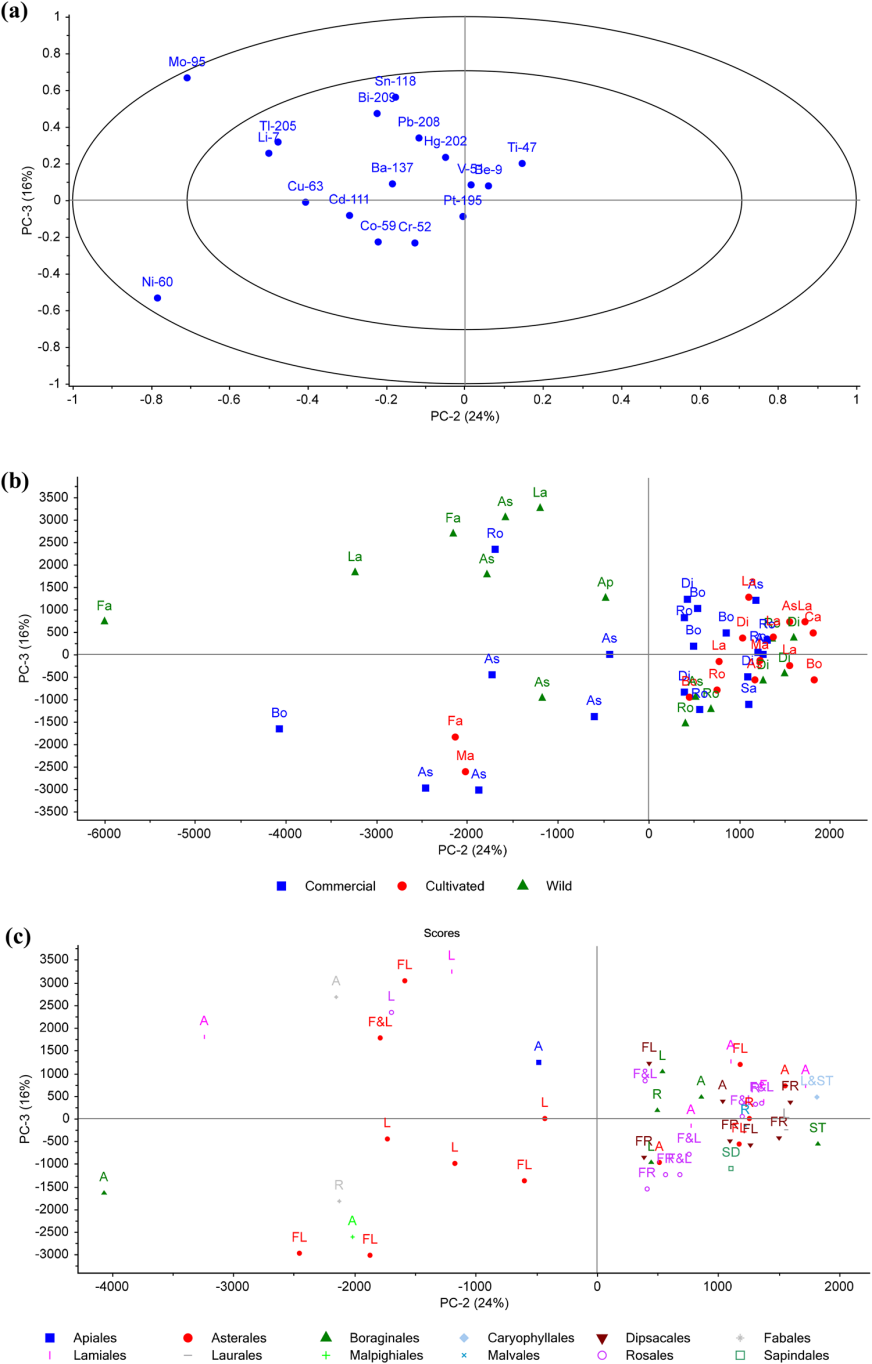
PCA (PC2vPC3) correlation loadings (**a**) and score plots grouped by (**b**) type and labelled by taxonomic order, and (**c**) grouped by taxonomic order and labelled by plant part. Taxonomic orders and plant part abbreviated as per Table 1.

The correlation loadings represent the element distribution (Figs. 2a and 3a). The elements in the outer percentile ring have a stronger influence on the score plots, which include Cr, Mo, Ni, Ti in Fig. 2a, and Mo and Ni in Fig. 3a. The score plots in Fig. 2b,c below are grouped by type and taxonomic order, and labelled by taxonomic order and plant part, respectively. PC1, PC2 and PC3 explain 35, 24 and 16% of the total correlation/variance, respectively.

As shown in Fig. 2b, there is a separation of the type category, the wild-collected samples group to the left side and the commercial to the right. A high concentration of Ti, V and Cr is observed in the commercial samples, independent of the taxonomic order. The cultivated samples are spread into both left and right quadrants which indicates a mix in quality/contaminants, independent of taxonomic order. It is plausible to suggest that contamination originating from the manufacturing process (i.e. post-harvest processing) may have contributed to the higher Cr content in these samples, compared to the cultivated and wild-collected counterparts. Similar findings have been reported in the literature for Cr transfer from industrial equipment to plant-based products, such as tea leaf processing^12^.

As shown in Fig. 2c, very little trending is observed overall, with slight grouping of the Dipsacales taxonomical order (Elder samples; *n* = 7) in the upper left quadrant which indicates a lower elemental concentration in comparison to the other taxonomic orders. No trend was observed based on plant part.

As shown in Fig. 3b, the majority of cultivated and commercial samples group to the right side revealing lower Mo and Ni levels compared to some of the wild samples. In Fig. 3c, the majority of the Rosales, Boraginales, Dipsacales samples tend to fall to the right of PC2 and based on the correlation loadings this shows that there is less Ni and Mo present in comparison to the other taxonomic orders.

### Mathematical modelling of non-carcinogenic human risk

The following formulae are commonly used to estimate the non-carcinogenic human risk associated with the theoretical consumption of herbal/medicinal plants, in the context of multi-element exposure. Health risk assessment of contaminants evaluate the probability of occurrence of harmful [non-carcinogenic] effects over a defined period of time. Table 4 summarises the key input data required for the mathematical assessment (see Eqs. (1) to (4)): The Estimated Daily Intake (EDI), Chronic Daily Intake (CDI), (Target) Hazard Quotient ((T) HQ) and Hazard Index (HI).

#### Calculation of the estimated daily intakes (EDI) and chronic daily intakes (CDI)

##### Estimated daily intake (EDI)

The EDI (µg (kg BW)^−1^d^−1^) for each metal was calculated using the following Eq. (1) as per Chen et al.^13^ and Luo et al.^8^, with adaptions. As outlined in Table 4; “**C**” represents the analyte concentration detected per sample (mg kg^−1^) (see Table 5), “**IR**” represents the ingestion/intake rate (kg.day^-1^), and “**BW**” refers to the default adult body weight proposed by EFSA^14^ of 70 kg. Dosage information provided by the manufacturer/supplier can be input for the IR in this equation; however, for this study, the sample matrices are raw plant material (i.e., not in final “consumer-ready” format) and thus recommended daily intakes are not possible. Generic IRs of 200 and 500 g.day^-1^, signifying the mean and 95th percentile (maximum daily dosage) of Chinese Herbal Medicinal Products, respectively, was proposed in the 2020 Chinese Pharmacopeia^15^ and used in recent health risk assessments of herbal preparations^8,18^. In the absence of a validated European equivalent; 200 g.day^-1^ was surrogated in this study to represent a theoretical maximum or “conservative” IR^15^ and a theoretical minimum IR of 200 mg.day^-1^^13^ to represent a more “realistic” exposure scenario.

**Table 4:** List of input data for the evaluation of exposure and risk (Eqs. (1) to (4)).

Input parameter(s)	Abbreviation	Value		Unit(s)	References
Estimated daily intake	EDI	–		mg (kg BW)^−1^d^−1^	^8,13^
Concentration	C	–		mg kg^−1^	^8,13^
Body weight (adult; European default value)	BW	70		kg	^14^
Ingestion/intake rate	IR	Low	0.0002	kg day^−1^	^13^
		High	0.2		^15^
Exposure frequency	EF	90		days	^8,13^
Exposure duration	ED (shorter-than-lifetime)	20		years	^8,13^
	ED (average lifetime)	70		years	
Transfer rate (metal)	*t*	Cd Cu Pb	14	%	^8,13^
		Hg	24		
		All other elements	10		
Average exposure time (average lifetime (ED) = 365 days × *70 years)*	AT	25,550		days	^8,13^
Reference dose (oral)	R*f*D	Cd (1.00 × 10^–3^) Cr (3.00 × 10^–3^) Cu (4.00 × 10^–2^) Mn (4.6 × 10^–2^) Ni (2.00 × 10^–2^) Pb (3.50 × 10^–3^)		mg kg^−1^ day^−1^	^11,16^
Oral carcinogenic slope factor (SF)	SF	Cd (0.38) Cr (0.5) Pb (8.5 × 10^–3^) Ni (0.84)		mg kg^−1^ day^−1^	^16,17^

**Table 5:** Concentration (µg kg^−1^)* of each element in sample (BT-1 to -50) following (HR) ICP-SFMS multi-elemental analysis.

Sample ID # (BT-)	Element concentration (µg kg^−1^; ppb)																
	Li	Be	Mo	Cd	Sn	Ba	Pt	Hg	Tl	Pb	Bi	Ti	V	Cr	Co	Ni	Cu
1	1459.04	47.28	584.02	68.11	50.85	NR	5.85	29.87^*^	8.87	4248.07^*^	29.64^*^	NR	662.35	3056.50	313.18	1094.01	4457.41
2	206.89	13.39	466.29	121.38	33.89	NR	10.78	15.76	8.05	749.38	5.00	3587.99	297.49	1813.12	151.94	1000.03	NR
3	206.44	5.69	439.58	51.58	119.28	NR	32.68^*^	24.05	4.08	189.35	6.41	842.34	390.83	1538.83	429.85	1117.59	NR
4	56.54	4.45	104.26	39.50	10.95	NR	10.52	13.57	3.06	46.05	3.63	130.96	23.22	126.84	107.91	1606.71	NR
5	150.19	6.88	650.40	63.93	63.51	NR	32.61	12.75	4.41	250.31	8.10	3360.29	311.66	1206.86	183.51	704.07	NR
6	75.54	3.97	120.18	27.37	10.83	NR	8.90	6.15	5.34	87.09	3.10	894.11	59.19	1497.10	87.19	1447.82	4495.71
7	75.55	3.41	123.01	16.47	11.98	NR	23.86	9.18	2.94	41.64	2.80	360.53	37.48	2109.85	99.48	1531.64	4285.69
8	197.83	9.03	286.40	214.18	22.22	NR	12.68	8.95	4.24	738.37	6.09	1732.72	191.22	2077.63	240.86	NR	NR
9	234.95	12.99	257.01	186.38	16.15	NR	15.39	6.92	19.07	202.06	3.83	5827.41^*^	436.27	2112.83	101.32	1578.06	NR
10	895.25	121.50^*^	1441.35	325.33^*^	165.15^*^	NR	29.85	15.59	12.20	1283.91	17.50	NR	1442.37	NR	323.34	1568.96	4499.09
11	56.24	4.12	175.73	9.65	14.86	NR	14.33	11.44	2.92	82.12	3.43	952.26	65.38	425.96	29.39	864.28	NR
12	214.39	13.87	1883.17	21.81	20.56	NR	7.34	10.31	6.59	437.26	5.61	3938.04	389.71	898.59	111.43	1106.25	NR
13	79.30	6.00	273.62	8.71	15.23	NR	12.18	8.03	4.00	323.77	3.71	1713.62	173.59	908.34	63.09	1057.01	NR
14	37.46	3.38	922.31	10.35	15.60	1661.43	11.72	9.40	3.07	26.20	3.42	1703.95	29.26	324.48	265.87	229.94	3534.14
15	38.09	3.13	196.71	4.75	9.09	4645.63^*^	8.45	5.86	2.64	34.12	2.90	194.67	31.58	259.53	20.68	324.13	3550.81
16	118.48	6.29	431.88	9.65	44.37	NR	9.06	7.89	4.00	197.71	4.65	1853.72	887.82	1198.27	118.82	1787.27	4066.09
17	381.39	42.94	1171.88	24.94	73.04	NR	19.11	5.94	4.04	970.03	4.18	NR	1094.17	1188.02	216.94	639.92	3379.73
18	17.51	2.95	194.28	6.80	18.08	746.66	6.50	4.67	3.23	46.17	2.42	206.08	21.83	1272.38	25.48	771.37	5113.65
19	310.00	11.53	4216.93	24.71	27.51	NR	13.77	13.53	7.46	1006.34	4.81	3957.58	393.33	1316.26	128.42	2019.91	NR
20	364.45	13.14	259.08	37.26	30.12	NR	8.12	10.76	5.87	263.78	4.58	5273.93	686.60	1150.25	147.66	1200.46	4395.44
21	207.40	10.38	490.12	310.05	20.34	NR	13.07	11.58	5.29	292.46	3.23	3678.10	278.27	895.96	256.18	3715.12	NR
22	1042.02	61.27	568.64	115.43	< 0.60	NR	20.01	12.16	12.93	1410.54	12.03	4313.59	1757.80^*^	1631.93	318.70	1753.13	NR
23	936.02	31.08	1354.21	102.55	72.03	NR	25.90	16.31	8.77	815.06	6.73	NR	805.56	1483.01	154.77	1150.87	NR
24	187.63	12.37	291.01	41.82	12.75	NR	19.79	28.57	4.65	52.87	2.93	710.99	53.21	1347.84	80.92	1176.23	5345.06
25	124.55	4.45	74.62	43.23	6.78	NR	13.95	10.51	3.27	11.58	2.67	149.72	14.81	425.77	58.26	356.35	2010.36
26	800.76	18.23	2700.04	180.30	31.57	NR	15.06	16.33	34.51	491.45	6.99	NR	451.99	NR	177.94	5564.42	5238.88
27	576.92	28.83	929.22	193.85	45.13	NR	9.03	10.60	23.33	654.94	7.97	NR	581.08	2916.26	168.39	881.58	3891.23
28	926.04	36.35	280.04	72.54	18.75	NR	16.43	12.08	7.11	1022.82	9.15	NR	1136.53	3406.48	465.07	6060.33^*^	NR
29	NR	21.94	921.68	142.51	23.62	NR	7.85	11.62	12.18	738.85	6.57	2455.06	227.09	667.85	651.76^*^	3336.54	NR
30	58.96	5.47	NR	36.96	141.82	NR	10.46	9.46	7.27	279.96	13.65	720.18	75.05	191.28	60.11	868.67	6340.13^*^
31	3735.42	9.92	NR	47.97	34.61	NR	11.10	13.02	53.00	403.10	7.33	328.52	55.59	258.26	179.64	NR	5557.24
32	3964.03^*^	7.81	3718.31	171.12	122.02	NR	12.86	23.22	12.28	1128.09	11.10	1233.55	187.63	417.94	123.87	420.23	NR
33	405.66	4.19	1408.71	32.42	39.39	NR	10.65	7.20	11.28	243.78	7.45	649.65	61.10	178.83	92.69	3104.00	NR
34	117.73	8.02	2829.87	19.93	87.47	NR	9.79	15.89	6.62	433.22	9.13	904.84	124.57	290.08	30.10	1040.53	NR
35	1344.63	6.67	NR	111.52	33.16	NR	10.39	8.89	16.22	401.89	8.71	642.22	64.30	388.54	123.27	2688.47	NR
36	164.56	3.13	345.93	29.81	10.85	NR	2.05	11.71	3.02	54.31	5.92	221.46	20.19	99.64	59.96	1498.31	3904.57
37	478.57	7.29	3883.54	192.05	71.81	NR	8.01	14.55	5.87	514.85	13.72	2758.56	167.98	510.27	98.35	2239.53	NR
38	228.26	11.39	4504.50^*^	51.05	133.44	NR	8.59	13.83	90.82^*^	2047.83	28.37	4138.97	1023.23	1885.72	106.47	985.80	NR
39	911.24	11.18	411.12	51.82	31.58	NR	5.73	8.98	13.61	383.87	4.03	NR	370.05	871.08	78.19	628.80	NR
40	725.13	54.29	90.70	70.28	41.47	NR	6.73	17.67	11.26	1004.57	14.10	5500.88	629.59	1108.20	541.49	1177.67	1910.27
41	1044.78	51.59	430.67	12.33	59.08	NR	6.02	17.26	7.32	598.07	8.72	NR	953.66	1546.24	166.51	532.01	2318.00
42	617.44	16.91	1129.19	25.03	15.37	NR	7.32	8.72	25.17	167.88	3.50	NR	1117.97	2568.35	337.17	NR	3695.86
43	191.97	11.56	265.31	214.19	6.39	NR	9.09	6.80	7.85	474.40	4.65	3394.64	321.29	1012.27	135.85	1228.32	NR
44	405.54	22.74	447.92	8.64	35.94	NR	5.75	8.67	5.84	321.35	5.71	4323.13	381.75	1209.22	92.94	887.69	3746.49
45	100.32	5.04	728.99	129.62	12.45	NR	8.24	8.62	4.87	194.33	3.04	1238.44	138.19	534.89	275.12	NR	NR
46	301.43	14.87	1292.97	24.54	53.87	NR	7.89	21.74	5.93	732.89	6.38	4282.37	376.90	549.99	93.86	724.70	NR
47	566.17	4.67	373.96	19.04	10.41	1817.19	7.47	6.77	3.56	48.01	3.00	576.06	62.61	190.89	43.64	879.70	NR
48	241.70	8.39	928.82	23.39	30.85	NR	4.58	5.36	5.73	307.48	3.54	2205.42	159.13	608.89	55.20	717.33	NR
49	562.12	22.55	495.47	8.07	21.73	NR	9.65	15.20	4.64	259.96	4.48	1921.74	473.70	1286.46	113.17	782.23	4905.02
50	181.91	20.90	446.21	11.76	28.69	NR	8.61	15.72	3.50	129.26	3.21	NR	292.01	4534.43^*^	50.81	678.60	2408.17

NR represents the element concentrations which exceeded the highest calibration standard and could not be accurately quantified. The concentrations of Al, Au, Fe, Mg, and Mn in all samples tested were outside the calibration range and could not be accurately determined; and thus, they are excluded from this table. *Highest quantified concentrations of the corresponding element(s) are highlighted in bold.

1 $$EDI=\frac{C\times IR}{BW}$$

##### Chronic daily intake (CDI)

The CDI (shorter-than lifetime exposure scenarios)^19^ was estimated by inputting the relevant data outlined in Table 4 into the following Eq. (2) recommended by the U.S. EPA (2011)^20^ with adaptions^18^. Estimated EDI and CDI values were compared to current dietary intake limits, where available, as shown in Table 6.

**Table 6:** Summary of the range of results (minimum to maximum) for short-term Estimated Daily Intake (EDI) and Chronic Daily Intake (CDI) for each metal across all samples (*n* = 50) representing the worst-case exposure scenario at a high theoretical ingestion rate (i.e. 200 g day^−1^^15^) of medicinal herbs or preparations and corresponding limits.

Element	High EDI range (µg (kg BW)^−1^d^−1^)		High CDI range (µg (kg BW)^−1^d^−11^)		(Provisional)Reference oral dose ((p)R*f*D)/European dietary limits		
	Min	Max	Min	Max	(µg (kg BW)^−1^d^−1^)		References
Li	0.05^*^	11.33^*^	< 0.00	0.08	pR*f*D	2.0	^29^
Be	0.01	0.35	< 0.00	< 0.00	TDI^d^	2.0	^36^
Mo	0.21^*^	12.87^*^	< 0.00	0.09	UL	10.0	^30^
Cd	0.01	0.93	< 0.00	0.01	TWI	0.0025^a^	^37^
Sn	0.02	0.47	< 0.00	< 0.00	TWI	14.0^a^	^30^
Pt	0.01	0.09	< 0.00	< 0.00	N/A		
Hg (inorganic)	0.01	0.09	< 0.00	< 0.00	TWI	0.004	^38^
Tl	0.01^*^	0.26^*^	< 0.00	< 0.00	pR*f*D	0.08	^31^
Pb	0.03^*^	12.14^*^	< 0.00	0.12	BMDL^b^	0.5–1.5	^32^
Bi	0.01	0.08	< 0.00	< 0.00	NOAEL	1000^**c**^	^39^
Ti	0.37	157.17	< 0.00	1.11	N/A		
V	0.04	5.02	< 0.00	0.04	R*f*D	9.0	^40^
Cr	0.28	12.96	< 0.00	0.09	TDI^d^	300^e^	^41^
Co	0.06^*^	1.86^*^	< 0.00	0.01	TDI	1.4	^34^
Ni	0.66^*^	17.32^*^	< 0.00	0.12	TDI	13.0	^35^
Cu	5.46	18.11	0.05	0.18	AI	1.6^f^	^42^

Calculation of each individual analytical results not shown in table. Element(s) highlighted in bold(*) are in exceedance of accompanying dietary limits. ^a^Tolerable weekly intake (TWI) = mg (kg BW)^−1^wk^−1^; ^b^Benchmark dose levels (BMDL’s) for Pb (µg (kg BW)^−1^d^−1^): 0.5 (BMDL_01_ [Developmental neurotoxicity]), 1.5 (BMDL_01_ [blood pressure]) and 0.63 (BMDL_10_ [kidneys])^33^; ^c^No adverse effect level (NOAEL) = mg (kg BW)^−1^d^−1^; ^d^Tolerable daily intake (TDI); ^e^TDI for Cr(III); ^f^Average intake (AI) = mg day^−1^.

2 $$CDI= \frac{C\times IR\times EF\times ED\times t}{AT\times BW}$$

#### Hazard quotient (HQ)

The non-carcinogenic risk associated with consumption of the botanical ingredients/products under investigation was characterised by estimating the theoretical target hazard quotient (HQ). The HQ is a unitless ratio of exposure to a potentially harmful substance (i.e. EDI) over a specified period and the level at which no adverse effects are expected (i.e. reference dose (R*f*D))^21^. HQ < 1 indicates that no risk is expected, while HQ ≥ 1 indicates a potential health risk for consumers. HQ is a deterministic risk-assessment expression that allows a level of risk to be demonstrated but cannot estimate probabilistic risks to exposed populations above the maximum threshold^19^. The U.S. EPA^20^ proposed evaluation of the HQ by dividing exposure by the relevant R*f*D, as shown in Eq. (3) below. ISO oral R*f*D’s for traditional Chinese medicines (TCM) were used in this equation as per Luo et al.^8^ (mg kg^−1^ day^−1^): **Cd** (0.0005); **Cu** (0.04); **Hg** (0.0003); **Pb** (0.0035).

3 $$HQ=\frac{Exposure\, (CDI \; or \; EDI)}{RfD}$$

#### Hazard index (HI)

HI is defined as the sum of HQ obtained, as shown in Eq. (4)^16,20^. The HI was established to evaluate human health risk to exposure to more than one element at a time, i.e. simultaneous or cumulative exposure. Similarly, HI < 1 is considered ‘no risk’; conversely, HI ≥ 1 is considered as a ‘potential non-carcinogenic risk’. Ultimately, the higher the HQ or HI the greater the risk to consumers.

4 $$HI=\Sigma (HQ)$$

## Results and discussion

### Multi-elemental compositions and statistical analysis

This is the first multi-elemental analysis of arnica, bush vetch, sweet cicely, yellow rattle, bogbean, rock-tea and tufted catchfly to-date, to the best of our knowledge. A diverse range of concentrations were quantified for each element in this present study: **Li** (17.51–3964.03 µg kg^−1^); **Be** (2.95–121.50 µg kg^−1^); **Mo** (74.62–4504.50 µg kg^−1^); **Cd** (4.75–325.33 µg kg^−1^); **Sn** (6.39–165.15 µg kg^−1^); **Ba** (746.66–4645.63 µg kg^−1^); **Pt** (2.05–32.68 µg kg^−1^); **Hg** (4.67–29.87 µg kg^−1^); **Tl** (2.64–90.82 µg kg^−1^); **Pb** (11.58–4248.07 µg kg^−1^); **Bi** (2.42–29.64 µg kg^−1^); **Ti** (130.96–5827.41 µg kg^−1^); **V** (14.81–1757.80 µg kg^−1^); **Cr** (99.64–4534.43 µg kg^−1^); **Co** (20.68–651.76 µg kg^−1^); **Ni** (229.94–6060.33 µg kg^−1^) and **Cu** (1910.27–6340.13 µg kg^−1^). Au, Mg, Mn, Al, and Fe (data not shown) were outside the calibration range and could not be quantified (i.e. “NR”: not reported). Levels of ^137^Ba, ^7^Ti, and ^63^Cu were above the calibration range for 92%, 78% and 48% of all samples, respectively. The variability of the element profiles observed between samples, regardless of botanical relatedness, is not considered unusual^22^ considering that the elemental composition of plant tissues and organs is largely influenced by edaphic conditions, soil geochemistry and eco-physiological factors^23^. This terrestrial diversity consequently complicates the exploration of novel trends in diverse datasets using chemometrics.

Nevertheless, chemometric models can be powerful in the analysis and interpretation of metallomic data, and has been shown to differentiate plant species, manufacturers (sources) and geographical origin^24^. Milani et al.^25^ also demonstrated the association of certain elements with different herbal teas using PCA. Larger sample sizes and increased number of replicates can enhance chemometric outputs.

Based on our statistical analysis, PCA shows trends between the category for type, where the commercial samples have a higher amount of Ti, V and Cr present, independent of the taxonomic order. Renna et al.^26^ showed that plant species was a more critical discriminating factor than sampling location. On the contrary, relative trends based on geno- or pheno-types of the same plant species or the same botanical family, independent to the sampling locations, were described previously following ICP-OES (-optical emission spectrometry) and PCA^26^. When observing groupings of the taxonomic order very little trending was observed except that the Dipsacales order had a lower elemental concentration in comparison to the other taxonomic orders. Wild samples tend to have higher amounts of Mo and Ni, while Rosales, Boraginales, Dipsacales orders have less Ni and Mo. It is unclear whether the commercial samples were originally derived from a rural or (sub)urban setting. Suburban pollution (e.g. intensive exhaust emissions) has been shown to influence the element profiles of plants^27^. In addition to increased sample sizes (i.e. same family, same species), having greater control over the geographical origin and level of processing is an important consideration for future studies in this area.

### Output of the mathematical non-carcinogenic risk assessment

All of the samples analysed (*n* = 50) were below the compendial ML’s for metal impurities in herbal starting material/substances laid out in the European Pharmacopeia^28^: Cd (≤ 1 mg kg^−1^), Hg (≤ 0.1 mg kg^−1^), and Pb (≤ 5 mg kg^−1^). Furthermore, when compared to European dietary limits, all calculated chronic dietary exposure estimates (e.g. CDIs; see Table 6) are well within the acceptable ranges from an oral dietary perspective. All estimated daily intakes (EDI’s) derived from the lower IR (200 mg day^−1^) are well below established regulatory limits and so too are the majority of the EDI’s derived from the higher IR (200 g day^−1^); thus, dietary exposure to the analysed botanicals at the theoretical intakes used in this study, are of negligible concern to consumers with respect to Be, Cd, Sn, Hg, Bi, V, Cr, Cu exposure. As shown in Table 6 (represented in bold), however, levels of Li, Mo, Tl, Pb, Co, and Ni at higher intake rates may be of potential concern to consumers with regards to frequent or prolonged usage.

An EDI range of 0.05–11.33 µg (kg BW)^−1^d^−1^ was calculated for **Li**, which exceeds the provisional R*f*D (pR*f*D) of 2 µg (kg BW)^−1^d^−1^^29^. An EDI range of 0.21–12.87 µg (kg BW)^−1^d^−1^ was calculated for **Mo**, which exceeds the UL of 10 µg (kg BW)^−1^d^−1^^30^. An EDI range of 0.01–0.26 µg (kg BW)^−1^d^−1^ was calculated for Tl, which exceeds the pR*f*D of 0.08 µg (kg BW)^−1^d^−1^^31^. An EDI range of 0.03–12.14 µg (kg BW)^−1^d^−1^ was calculated for **Pb**, which exceeds the BMDL’s for Pb, which are (µg (kg BW)^−1^d^−1^): 0.5 (BMDL_01_ [Developmental neurotoxicity]), 1.5 (BMDL_01_ [blood pressure]) and 0.63 (BMDL_10_ [kidneys])^32,33^. An EDI range of 0.06–1.86 µg (kg BW)^−1^d^−1^ was calculated for **Co**, which exceeds the TDI of 1.4 µg (kg BW)^−1^d^−1^^34^. And lastly, an EDI range of 0.06–17.32 µg (kg BW)^−1^d^−1^ was calculated for **Ni**, which exceeds the recently updated TDI of 13 µg (kg BW)^−1^d^−1^^35^. Interstudy comparison to previous studies in the literature was challenging in the absence of standardisation or official guidance, i.e. variations in methodology, equations and input criteria, as discussed later.

Results from the mathematical non-carcinogenic exposure assessment are presented in Table 7. At the lower intakes level, consumption of the plants under investigation are considered safe and of no risk to consumers, with regards to Cd, Cu, Hg and Pb exposure (HQ and HI < 1). At a theoretical “worst-case” exposure scenario of 200 g day^−1^, as recommended by the Chinese Pharmacopeia^15^, 16% (Cd) and 8% (Pb) of samples are considered potentially unsafe (HQ ≥ 1). The plants indicating risk of Cd exposure include arnica, dandelion, yarrow, borage, mugwort and marshmallow. While plants indicating risk of Pb exposure include hawthorn, dandelion, comfrey and great mullein. Conversely, Cu and Hg exposure, even at a high ingestion rate, are still considered safe and of no risk to consumers. This is expected for Hg since levels are generally low in terrestrial plants^2^, however the results for Cu may not be entirely representative per se considering that 48% of the samples were above the calibration range, could not be quantified, and were therefore excluded from the risk assessments. This resulted in a smaller sample size for Cu.

**Table 7:** Theoretical Hazard Quotient (HQ) and resulting Hazard Indices (HI) estimated for Cd, Hg, Pb, and Cu—representing the worst-case exposure scenario only (i.e. Short-term EDI @ 200 g day^−1^ consumption).

Sample I.D.	HQ_[High EDI]_				HI_[High EDI]_
	HQ_(Cd)_	HQ_(Hg)_	HQ_(Pb)_	HQ_(Cu)_	
BT_01	0.39	0.28	3.47^*^	0.32	4.46^*^
BT_02	0.69	0.15	0.61	–	1.46^*^
BT_03	0.29	0.23	0.15	–	0.68
BT_04	0.23	0.13	0.04	–	0.39
BT_05	0.37	0.12	0.20	–	0.69
BT_06	0.16	0.06	0.07	0.32	0.61
BT_07	0.09	0.09	0.03	0.31	0.52
BT_08	1.22^*^	0.09	0.60	–	1.91^*^
BT_09	1.07^*^	0.07	0.16	–	1.30^*^
BT_10	1.86^*^	0.15	1.05^*^	0.32	3.38^*^
BT_11	0.06	0.11	0.07	–	0.23
BT_12	0.12	0.10	0.36	–	0.58
BT_13	0.05	0.08	0.26	0.26	0.65
BT_14	0.06	0.09	0.02	0.25	0.42
BT_15	0.03	0.06	0.03	0.25	0.36
BT_16	0.06	0.08	0.16	0.29	0.58
BT_17	0.14	0.06	0.79	0.24	1.23^*^
BT_18	0.04	0.04	0.04	0.37	0.49
BT_19	0.14	0.13	0.82	–	1.09^*^
BT_20	0.21	0.10	0.22	0.31	0.84
BT_21	1.77^*^	0.11	0.24	–	2.12^*^
BT_22	0.66	0.12	1.15^*^	–	1.93^*^
BT_23	0.59	0.16	0.67	–	1.41^*^
BT_24	0.24	0.27	0.04	0.38	0.94
BT_25	0.25	0.10	0.01	0.14	0.50
BT_26	1.03^*^	0.16	0.40	0.37	1.96^*^
BT_27	1.11^*^	0.10	0.53	0.28	2.02^*^
BT_28	0.41	0.12	0.83	–	1.36^*^
BT_29	0.81	0.11	0.60	–	1.53^*^
BT_30	0.21	0.09	0.23	0.45	0.98
BT_31	0.27	0.12	0.33	0.40	1.12^*^
BT_32	0.98	0.22	0.92	–	2.12^*^
BT_33	0.19	0.07	0.20	–	0.45
BT_34	0.11	0.15	0.35	–	0.62
BT_35	0.64	0.08	0.33	–	1.05^*^
BT_36	0.17	0.11	0.04	0.28	0.61
BT_37	1.10^*^	0.14	0.42	–	1.66^*^
BT_38	0.29	0.13	1.67^*^	–	2.10^*^
BT_39	0.30	0.09	0.31	–	0.69
BT_40	0.40	0.17	0.82	0.14	1.53^*^
BT_41	0.07	0.16	0.49	0.17	0.89
BT_42	0.14	0.08	0.14	0.26	0.63
BT_43	1.22^*^	0.06	0.39	–	1.68^*^
BT_44	0.05	0.08	0.26	0.27	0.66
BT_45	0.74	0.08	0.16	–	0.98
BT_46	0.14	0.21	0.60	–	0.95
BT_47	0.11	0.06	0.04	–	0.21
BT_48	0.13	0.05	0.25	–	0.44
BT_49	0.05	0.14	0.21	0.35	0.75
BT_50	0.07	0.15	0.11	0.17	0.49

Values highlighted in bold (*) represent exceedance of HQ and HI ≥ 1 (i.e., “potentially unsafe”). Values < 1 considered “no risk” or “safe”.

HQ’s calculated at the lower EDI (@200 mg day^−1^) and both CDI’s (@200 mg day^−1^ and g day^−1^) were HQ ≤ 0.01 and HI ≤ 0.05, respectively, and thus considered of “no risk” to consumers. These values are therefore excluded from this table (data not shown).

A total of 42% of samples are categorised as potentially unsafe (HI ≥ 1) regarding cumulative exposure to Cu, Cd, Hg and Pb, representing 16 different plant species in total. From these calculations, oral consumption of the following plants could potentially cause non-carcinogenic health risks to consumers: hawthorn, arnica, dandelion, marigold, nettle, yarrow, comfrey, borage, coltsfoot, birds-foot trefoil, ox-eye daisy, yellow rattle, mugwort, great mullein, tufted catchfly and marshmallow.

### Critical discussion of the analysed plants

The following section covers in-depth the quantified element concentrations in each plant species (*n* = 30) with comparison to reported elemental profiles in the literature (where available), and the results of the subsequent non-carcinogenic risk assessments performed.

#### *Crataegus laevigata* (hawthorn)

Element concentrations appeared lower in hawthorn fruit/berry samples (BT-06 and 07) than the flower and leaf (BT-01 to -05); however a comparison to the available literature was not possible since the leaf and flower samples analysed were acquired pre-mixed, and previous studies^43^ analysed flower, leaf, berry and seed separately. Our findings for Cd, Co, Cr, Cu are largely within the ranges reported; while differences were noted for Li, Mo, Ni^43^ and V^44^.

Hawthorn flower and leaf (BT-1) sourced from China presented with the highest non-carcinogenic risk (HI = 4.46) out of all the samples tested (see Table 7), based on the EDI at an IR of 200 g day^−1^, as recommended by the Chinese Pharmacopeia^15^. The same sample contains potentially unsafe levels of Pb (HQ_(Pb)_ = 3.47) at a quantified concentration of 4248.07 µg kg^−1^, which is 300 times higher than the lowest concentration detected in this study (BT-25; comfrey stem), and much higher than data previously reported in similar studies for the flowers^43^ and fruits^44^. Additionally, BT-1 presented with the highest Bi levels out of all the samples tested, 29.64 µg kg^−1^—although at this concentration, no risk (HQ < 1) was determined at an EDI and CDI of 0.014 and < 0.0001 µg (kg BW)^−1^d^−1^, respectively. PCA revealed that Pb and Bi strongly occurred together, as shown in Fig. 2. Both Pb and Bi—in addition to primordial and anthropogenic-derivatisation—are also daughter isotopes of nuclear decay chains (e.g. actinium, thorium, uranium/radium series). Their presence in aerial samples of hawthorn, if theoretically derived from radionuclide decay in soil matrices, could indicate the successful root to aerial transfer of these elements *in planta*. There is no experimental data in the literature currently to support this.

The same sample (BT-1) also presented with the highest Hg concentration (29.87 µg kg^−1^) out of all the samples tested. No risk was detected for the oral consumption at both high and low theoretical exposure scenarios investigated (HQ_(Hg)_ and HI < 1). The sample also contained levels of Ti above the calibration range, and therefore could not be quantified—while the hawthorn sample wild-collected in Co. Cork (Ireland) (BT-4) contained the lowest Ti levels out of all the samples tested. Hawthorn flower and leaf (BT-2) sourced from Eastern Europe (BT-2) also presented as a potential non-carcinogenic risk for cumulative exposure to Cu, Cd, Hg, and Pb (HI = 1.46), based on the EDI derived from the higher IR. Furthermore, an aerial (flower and leaf) sample collected from a farm in West Cork (Ireland) (BT-3) presented with the highest Pt level (32.68 µg kg^−1^) out of all the samples tested. No risk was detected for the oral consumption at both high and low theoretical exposure scenarios investigated (HQ’s and HI < 1). The remaining hawthorn samples (BT-2 to 7) were also considered safe and of no risk to consumers, based on the results of the exposure assessments detailed earlier.

#### *Arnica montana* (Arnica)

Our findings are, to the best of our knowledge, the first account of the multi-elemental profiling of *Arnica* spp. using (HR) ICP-SFMS. Levels of Ba, Ni, and Cu were not quantifiable in arnica flowers. Based on the results of the exposure assessment using EDI’s derived from the higher IR (see Table 6), a potential risk of unacceptable Cd exposure (HQ = 1.22) and subsequent risk of cumulative exposure to Cd, Cu, Hg, Pb (HI = 1.91) was detected for the oral consumption of arnica flowers (BT-8). Conversely, no risk was detected when considering the EDI derived from the lower IR, and CDI’s.

#### *Taraxacum officinale* (dandelion)

Dandelion is known to preferentially accumulate metals in aerial tissues rather than underground organs^45^, which was reflected in our results where we observed highest levels of Be (121.50 µg kg^−1^), Cd (325.33 µg kg^−1^) and Sn (165.15 µg kg^−1^) across all fifty samples in certified-organic dandelion leaf (BT-10) sourced from the Netherlands. An exception was Ti (5827.41 µg kg^−1^) in organic root from France (BT-9), which exhibited the highest Ti concentrations across all samples—however, the Ti content in the leaf could not be quantified as it was above the calibration range. From the results of the exposure assessment, dandelion leaf (BT-10) represents the second highest non-carcinogenic risk (HI = 3.38) out of all the samples tested (see Table 7), based on the EDI at an IR of 200 g day^−1^. The same sample also contains potentially unsafe levels of Cd (HQ_(Cd)_ = 1.86) and Pb (HQ_(Pb)_ = 1.05). These results are in accordance with Luo et al.^8^ who reported an EDI_(Pb)_ of 4 µg kg^−1^ day^−1^ for dandelion aerial parts at a higher IR (0.5 kg day^−1^), and a HI > 1. The root (BT-9) contains potentially unsafe levels of Cd too (HQ_(Cd)_ = 1.07), and a cumulative risk of toxicity to consumers (HI = 1.30).

Our findings did not demonstrate higher accumulation of Cd than Ni as previously reported^46^ but were within the lower ranges (< 0.0004 and < 0.0002 µg kg^−1^) reported by Lisiak-Zielinska et al.^47^. We observed similar Cu^46^, but lower Cr levels than previously reported^47^. Another study^48^ described a benchmark level of 200 µg kg^−1^ for Pb in dandelion leaves—a concentration that is more in line with the root (BT-09; 202.06 µg kg^−1^) compared to the elevated leaf concentration (BT-10; 1283.91 µg kg^−1^) in this study. Levels of Pt and Pb are more reflective of those reported previously^49^. The authors observed the accumulation of traffic emission-related Platinum Group Elements (PGE’s) in dandelion, with profiles positively correlated with the PGE pollution profile of environmental street dust sampled at the same time. The increased prevalence of PGE’s in plants growing along motorways since the introduction of catalytic converters was acknowledged^49^, leading to further research focusing on dandelion as a promising biomonitoring and remediation tool for urban environmental pollution^46^. Studies concerning the phytoextraction of rare earth elements (REEs) using dandelion^50^ and other species is increasing and is likely to continue to do so considering the projected global initiatives supporting widespread use of electric motor vehicles; the design of which utilises REE’s in current models.

#### *Sambucus nigra* L., *S. nigra *flos and *S. nigra* fruct. (elder, elderflower and elderberry)

Wild-collected elderberry fruit (BT-14) contained the lowest levels of Ni. Another wild-collected elderberry sample (BT-15) from Co. Cork (Ireland) contained notably lower concentrations of Cd (4.75 µg kg^−1^), Tl (2.64 µg kg^−1^), and Co (20.68 µg kg^−1^) compared to the other samples; and the highest quantified concentration of Ba (4700 µg kg^−1^). Ninety-two percent of the samples tested were above the calibration range for Ba and consequently could not be quantified, and therefore this is not entirely representative of the sample set per se. Similar levels of Cd and Hg were reported by Schulzki et al.^51^ compared to an average Cd and Hg concentration of 12.6 and 8.5 µg kg^−1^, respectively, across all elder-derived samples in this study (BT-11 to 16 (flower and fruit) and BT-48 (Spanish Elder)). Pace et al.^52^ observed higher Pb levels in aerial tissues (8700–13,700 µg kg^−1^) compared to the fruit (900 µg kg^−1^) of elder sampled from a Pb-contaminated site. Our results demonstrate a wide variation in Pb concentration among the elder-derived samples (26–437 µg kg^−1^), with levels in wild-collected samples from Co. Cork (Ireland) measurably lower than commercial and cultivated counterparts. Based on the output of the exposure assessment (see Table 7), no risk was detected for the oral consumption of any of the elder-derived samples when considering both the high and low theoretical exposure scenarios modelled in this study (i.e. 200 mg day^−1^ and 200 g day^−1^).

#### *Calendula officinalis* (marigold)

Studies on the phyto-remedial potential of *C. officinalis* seedlings^53^, hydroponic cultures^54^, and the aerial phytostabilization of Cd by *C. officinalis*^55,56^ and related species *C. calypso*^57^, has been explored; yet the multi-element analysis of marigold raw materials appears infrequently in the literature. Levels of Ba and Ti were above the calibration range and could not be quantified. This current study revealed a potential risk of cumulative exposure to Cd, Cu, Hg, Pb (HI = 1.23) from marigold flower consumption based on the EDI derived from the higher IR (200 g day^−1^).

Levels of Pb (970.03 µg kg^−1^) in the flowers were measurably lower than those previously reported in the inflorescences (93,400 µg kg^−1^) and leaves (11,570 µg kg^−1^) collected near a motorway. The proximity to the motorway may have influenced the higher Pb levels observed by Meos et al.^58^—who further advised against the collection of the leaf [for subsequent analysis] during or directly after rain showers. In agreement, Deljanin et al.^59^ observed a 30% reduction in Pb load after rinsing plant material before analysis. These are examples of collection parameters that could be considered, for example, in the WHO GACP guidelines or equivalent.

#### *Aesculus hippocastanum* (horse chestnut)

Several studies in the literature report the analysis of horse chestnut leaf composition^60,61^; yet few regarding seed composition exist. Levels of Li, Be, Ba, Hg, Bi were lowest in horse chestnut seed (BT-18) out of all the samples analysed in this study. Levels of Ni in seed (BT-18) sourced from the UK were comparable to those reported^62^, however Cr was notably higher, and Cu much lower in our study. Levels of Cd, Hg and Pb were also much lower compared to those previously reported^63^. Based on the output of the exposure assessment (see Table 7), no risk was detected for the oral consumption of horse chestnut seed when considering both the high and low theoretical exposure scenarios modelled in this study (i.e. 200 mg day^−1^ and 200 g day^−1^).

#### *Urtica dioica* (nettle)

Nettle leaf and root are reportedly good biomonitoring indictors for Cr, Pb and Zn^64^. Our findings for both nettle samples (BT-19 to 20) were lower for Cd, Cr, Tl and Pb, and within the lower range of concentrations for Co, Ni and V reported previously^65^. Hg concentrations are comparable to Fischer et al*.*^66^. In agreement with Mihaljev et al.^67^, higher Mo content was observed in the leaf (4216.93 µg kg^−1^) than root (259.08 µg kg^−1^) (see Table 5). Similarly, levels of Pb and Ni were higher in the leaf than root. Ba and Fe were poorly leached from nettle infusions^68^, indicating a poor transfer rate from this matrix. Based on the output of the exposure assessment (see Table 7), no risk was detected for the oral consumption of certified-organic nettle root (BT-19) when considering both the high and low theoretical exposure scenarios modelled in this study (i.e. 200 mg day^−1^ and 200 g day^−1^). Conversely, a potential non-carcinogenic risk for the cumulative exposure following consumption (i.e. high EDI) of the [non-organic] leaf was calculated (HI = 1.09).

#### *Achillea millefolium* (yarrow)

Yarrow can be utilised as a bioindicator and phytoremediator (As, Cd, Pb) in polluted soils^69^. Comparable Ni^70^ and Pb levels^71^ were observed in this study and lower Sn (20 vs 3000 µg kg^−1^) and Mo (490 vs 2300 µg kg^−1^)^67^. Elevated Cd (310 vs 76 µg kg^−1^), Co (256 vs 21 µg kg^−1^), and Cr (896 vs 490 µg kg^−1^) levels were observed in our analysis of yarrow flowers (BT-21) compared to concentrations in Zeiner et al.^72^. Based on the results of the exposure assessment using EDI’s derived from the higher IR (see Table 7), a potential risk of unacceptable Cd exposure (HQ_(Cd)_ = 1.77) and subsequent risk of cumulative exposure (HI = 2.12) was detected for the oral consumption of yarrow flowers (BT-21).

#### *Symphytum officinale* (comfrey)

Studies qualitatively described the composition of comfrey^73,74^, and the in vitro modelling of Pb-tannin chelation *in planta*^75^. In a cluster containing coltsfoot too, comfrey demonstrated measurably higher Fe levels than other medicinal plants, as well as Zn and Cr^73^. In this current study, the stem (BT-25) contained the lowest Mo, Pb, and V compared to all the samples analysed (*n* = 50). The commercial root (BT-22), conversely, had the highest quantified V level of all the samples. Based on the results of the exposure assessment using EDI’s derived from the higher IR (see Table 7), a potential risk of unacceptable Pb exposure (HQ_(Pb)_ = 1.15) for the consumption of comfrey root (BT-22) was detected. A subsequent non-carcinogenic risk of cumulative exposure was detected for the oral consumption of commercial root (BT-22) from Bulgaria, and leaf (BT-23) from Hungary (HI = 1.93 and 1.41). The remaining comfrey samples (BT-24 and 25) collected from a farm in Co. Cork (Ireland) contained notably lower Cd, Pb, Ti, V, and Co than the commercial samples and were considered safe for consumer consumption (HQ and HI < 1).

#### *Borago officinalis* (borage)

A recent study profiled the phytochemical composition of borage flowers, excluding mineral nutrients^76^. Another study^77^ reported higher Cu and Li concentrations compared to those quantified for BT-25 and -26 in this study. Based on the results of the exposure assessment using EDI’s derived from the higher IR (see Table 7), a potential risk of unacceptable Cd exposure was detected for the consumption of powdered (BT-26; HQ_(Cd)_ = 1.03) and cut (BT-27; HQ_(Cd)_ = 1.11) aerial material from Germany. A subsequent non-carcinogenic risk of cumulative exposure was detected for the oral consumption of both samples (BT-22, HI = 1.93; and BT-23, HI = 1.41).

#### *Tussilago farfara* (coltsfoot)

The hyperaccumulating potential of coltsfoot was recently investigated^78,79^. In a recent analysis of medicinal plants, coltsfoot contained the highest levels of Cr, Fe, K, Ni, and the lowest concentration of Pb^73^. In agreement, Petukhov et al.^80^ noted highest Fe accumulation in coltsfoot in addition to Mn and Zn, however since the concentrations of these elements were not quantified for these samples in this study, a comparison with the levels reported cannot be made. Comparable levels of Cd, Cr, Ni and Pb—in line with Wechtler et al.^79^—were observed for coltsfoot flowers from Albania (BT-28) and leaves from Poland (BT-29). The flowers contained the highest Ni (6060.33 µg kg^−1^) levels; and the leaves contained the highest Co (651.76 µg kg^−1^) levels out of all fifty samples analysed in this current study. Coltsfoot leaf (BT-29) was the only sample where Li was above the calibration range and could not be quantified. Based on the results of the exposure assessment using EDI’s derived from the higher IR (see Table 7), both samples displayed safe HQ values (i.e. < 1). The non-carcinogenic risk of cumulative exposure, however, was detected for the oral consumption of both samples (BT-28, HI = 1.36; and BT-29, 1.53). These results are in-agreement with Luo et al*.*^8^ who reported HI > 1 for coltsfoot flower.

#### *Vicia sepium* (bush vetch)

This is the first multi-elemental analysis of bush vetch to the best of our knowledge. Other studies have investigated related species, for example the in situ phytostabilisation of Cd, Pb and Zn in *Vicia sativa*^81^, induced Hg accumulation in *Vicia villosa* (hairy vetch) (Moreno et al., 2005) and accumulated levels of Cu, Fe, Pb and Zn in *Vicia cracca* (wild bird vetch)^80^. Based on the results from the mathematical risk assessment (see Table 7), there was no risk detected (i.e. HQ and HI < 1) for the oral consumption of aerial bush vetch wild-collected in Ireland, at the theoretical exposure scenarios modelled in this study (i.e. 200 mg day^−1^ and 200 g day^−1^), and thus these samples are considered safe.

#### *Lotus corniculatus* (birds-foot trefoil)

Babincev^82^ reported elevated levels of Pb (87,000–254,000 µg kg^−1^) and Cd (3000–11,000 µg kg^−1^) in birds-foot trefoil compared to our findings (BT-31) of 403.10 and 47.97 µg kg^−1^, respectively—otherwise, elemental data is limited in the literature for this plant. Based on the results of the exposure assessment using EDI’s derived from the higher IR (see Table 7), an aerial sample of birds-foot trefoil wild-collected in Ireland displayed safe HQ values (i.e. < 1). The non-carcinogenic risk of cumulative exposure, however, was detected for the theoretical oral consumption of the sample (HI = 1.12), at the higher IR.

#### *Leucanthemum vulgare* (ox-eye daisy)

An earlier study^83^ observed Pb and Zn accumulation in ox-eye daisy; however, no other information is available in the literature concerning its elemental composition. The highest Li concentration out of all fifty samples was observed in the flower (BT-32) at 3964.03 µg kg^−1^, which is over 200 times higher than the lowest value observed for horse-chestnut seed (BT-18). The flower (BT-32) typically contained higher levels of all elements analysed, except for Ni, when compared to the leaf (BT-33) sampled from the same parent plant which was wild-collected in Ireland. As a result, despite demonstrating safe HQ values (i.e. < 1), a non-carcinogenic risk of cumulative exposure was detected for the flower (BT-32, HI = 2.12) but not the leaf (see Table 7).

#### *Myrrhis odorata* (Sweet Cicely); *Rhinanthus minor* (Yellow Rattle); *Menyanthes trifoliata* (Bogbean); *Jasonia glutinosa L. (DC) (Rock Tea)*

This is the first multi-elemental analysis of sweet cicely, yellow rattle, bogbean and rock tea, to the best of our knowledge. Bogbean (BT-36; aerial) contained lowest levels of Pt (2.05 µg kg^−1^) and Cr (99.64 µg kg^−1^) out of all fifty samples analysed. Levels of Ti and Mo were above the calibration range and could not be quantified for yellow rattle and rock tea, respectively. Out of the 4 novel samples, Cu was only quantifiable in bogbean (BT-36; aerial) at 3904.57 µg kg^−1^. Based on the results from the mathematical risk assessment (see Table 7), there was no risk detected (i.e. HQ and HI < 1) for the oral consumption of the aerial samples of wild Irish sweet cicely (BT-34) and bogbean (BT-36), or rock tea from Spain (BT-39), at the theoretical exposure scenarios modelled in this study (i.e. 200 mg day^−1^ and 200 g day^−1^). A non-carcinogenic risk of cumulative exposure was detected for wild-collected yellow rattle from Ireland (BT-35; HI = 1.05), however, based on the EDI derived from the high IR (i.e. 200 g day^−1^) or “worst-case” exposure scenario.

#### *Artemisia vulgaris* (mugwort)

Elemental data for *A. vulgaris* (mugwort) is scarce, with reports limited to Cd-accumulation^84^ and other related species, i.e. *A. arborescens* (wormwood)^85^, *A. sphaerocephala*^86^, *A. fragrans*^87^ and *A. argyi* (Chinese mugwort)^88^. Levels of Al, As, Pb, Cd, Cu, Fe, Mn, Se, Zn in a Saudi-Arabian *Artemisia* product named “Sheeh” were reported by Brima^89,90^. Based on the results of the exposure assessment using EDI’s derived from the higher IR (see Table 7), a potential risk of unacceptable Cd exposure (HQ_(Cd)_ = 1.10) and subsequent risk of cumulative exposure (HI = 1.66) was detected for the oral consumption of mugwort flower and leaf (BT-37) wild-collected in Ireland.

#### *Verbascum thapsus* (great mullein)

There are several studies on the multi-elemental composition of related species *V. olympicum* Boiss.^91–93^, *V. bombyciferum* Boiss.^94^, *V. speciosum*^95,96^*, V. cheiranthifolium*^97^, *V. densifolium*^98^) and *V. phlomoides*^99,100^, however data is limited for *V. thapsus.* Recent studies have investigated *V. thapsus* Cd accumulation, Cu phytoextraction efficiencies^101,102^, morphological changes following Cd, Cr, Pb and Zn contamination^103^ and suitability as an Organisation for Economic Co-Operation and Development (OECD) test plant species^104^. Another study quantified Pb levels in the root and shoot (1342 and 995 mg.kg^−1^) that were much higher than our findings for BT-38 (2047.83 µg kg^−1^)^105^. Wild-collected great mullein from Co. Sligo (Ireland) contained highest levels of Mo (4504.50 µg kg^−1^) and Tl (90.82 µg kg^−1^) in this current study. Based on the results of the exposure assessment using EDI’s derived from the higher IR (see Table 7), a potential non-carcinogenic risk of unacceptable Pb exposure (HQ_(Pb)_ = 1.67) and subsequent risk of cumulative exposure (HI = 2.10) was detected for the oral consumption of great mullein leaves (BT-37).

#### *Silene saxifraga* L. (tufted catchfly)

Analyses of related species are found in the literature, such as Tl accumulation in *S. latifolia*^106^, As accumulation in *Silene vulgaris*^107^, and Cu tolerance in *S. paradoxa*^108^. This current study is however, the first multi-elemental analysis of this species. The lowest quantifiable levels of Cu were detected in tufted catchfly (BT-40) at 1910.27 µg kg^−1^. Based on the results from the mathematical risk assessment (see Table 7), there was no risk detected (i.e. HQ and HI < 1) for the oral consumption of the aerial leaf and stem samples of tufted catchfly (BT-40) at the theoretical exposure scenarios modelled in this study (i.e. 200 mg day^−1^ and 200 g day^−1^). A non-carcinogenic risk of cumulative exposure however was detected (HI = 1.53), based on the EDI derived from the high IR (i.e. 200 g day^−1^) or “worst-case” exposure scenario.

#### *Salvia officinalis* L. (sage)

Sage reportedly has both Cd and Pb-accumulating capacities^109^. Metals tend to accumulate in the aerial parts, including the inflorescences^110^. In previous studies, mean Cu content in sage leaf was 1400^111^ and 10,500 µg kg^−1^^112^ in comparison to 2318 µg kg^−1^ (BT-41) derived in this study. Our findings are similar for Co, lower for Cd, Ni, Pb, and higher for Cr and V when compared to Thabit et al.^113^. Levels of Cu, Cd and Pb were also lower than those reported in other studies using atomic absorption spectrometry AAS^114^ and flame atomic absorption spectrometry ( FAAS)^115^. There was no risk detected (i.e. HQ and HI < 1) for the oral consumption of aerial sage material sourced from Spain at the theoretical exposure levels modelled in this study.

#### *Glycyrrhiza glabra* (liquorice)

Elevated levels of Cr, Cu and Pb analysed via total reflection X-ray fluorescence (TRXF) were reported previously^116^. Similarly, higher levels were observed for Cd (720 vs 25.03 µg kg^−1^)^117^ and Li (1800 vs 600 µg kg^−1^)^118^ compared to our findings. Other studies analysed liquorice stem and leaf only which is outside the scope of this study^119^. There was no risk detected (i.e. HQ and HI < 1) for the oral consumption of liquorice root sourced from Spain at the theoretical exposure levels modelled in this study.

#### *Althaea officinalis* (marshmallow)

Elemental data is limited in the literature for this species, with few studies exploring the Cd, Cu, Pb and Ni accumulating potential of the related *A. rosea* Cavan^120^; which is an established Cd-tolerant species capable of accumulating Cd in the roots^121^. Our findings for Co and Ni in marshmallow root are in good accordance to the data published by Mihaljev et al.^67^—however, Mo and Sn content in BT-43 was lower. Sn concentration in the root (BT-43) was in fact the lowest out of all samples analysed in this current study (6.39 µg kg^−1^). Relatively high Cd, Cr, Ni, Pb and Ti levels were observed (see Table 5). Based on the results of the exposure assessment using EDI’s derived from the higher IR (see Table 7), a potential risk of unacceptable Cd exposure (HQ_(Cd)_ = 1.22) and subsequent non-carcinogenic risk of cumulative exposure (HI = 1.68) was detected for the oral consumption of the root (BT-43) sourced from Spain.

#### *Lavandula angustifolia* (lavender)

Lavender is a valuable essential oil crop. Our findings for Pb^122^, Cr and Ni^123^ are in line with previous results. Data for Co, Cu and V are measurably lower than previous reports^122,124^. Interestingly, Zheljazkov and Nielsen^125^ further observed a positive correlation between the Cu concentration in the inflorescence and the resulting essential oil derived—but not for Cd, Pb, Mn, Fe, Zn. This agrees with a later study of trace element transfer [flower to oil] in *L. angustifolia*^122^. Sierra et al.^126^ also describes lavender as a Hg tolerant excluder, and the presence of Hg and Mn influences Pb uptake in lavender. There was no risk detected (i.e. HQ and HI < 1; see Table 7) for the oral consumption of lavender flower sourced from Spain at the theoretical exposure levels modelled in this study.

#### *Hypericum perforatum* (St. John’s Wort)

St. John’s Wort is a popular medicinal plant. Element profiling using ICP-OES exists in the literature^24,111,127,128^, but is limited for ICP-MS^129^. This is one of the first multi-element ICP-MS analyses covering a large suite of key elements in St. John’s Wort. Our findings for Co, Cr^23^, Cd^130^ and Pt^24^ were lower than previously reported. Leaf Cu^111^, Zn^131^, Ni and Ba^130^ could not be compared as they were above the calibration range in this study and thus could not be quantified. Owen et al.^24^ reports that elevated Cr, Y (yttrium) and Sr (strontium) concentrations in St. John’s Wort medicinal products could be due to contamination from metal alloys in the manufacturing process. This reflects observations in this current study regarding increased Cr levels in commercially processed plant material. The herb is reportedly a Cd-accumulator, however, the leaching efficiency of Cd in herbal tea/infusion containing St. John’s Wort (aerial herb) was found to be low^132^. There was no risk detected (i.e. HQ and HI < 1) for the oral consumption of an aerial parts of St. John’s Wort (BT-45; see Table 7) sourced from Spain at the theoretical exposure levels modelled in this study, and thus deemed safe.

#### *Melissa officinalis* (lemon balm)

Our findings for Cr, Cu, Ti, V and Ni were lower than those reported previously^131,133,134^; except for Co^134^ and Pb^131^. Ward and Savage^135^ demonstrated that washing can remove 49% of Pb from lemon balm leaves. Cd-Zn interactions have been shown to alter Cu, Pb and Mn uptake in lemon balm^136,137^. Cd was shown to reduce essential oil yield in lemon balm seedlings^138^, thus demonstrating that cultivation parameters can impact the medicinal value of a plant. In this study, there was no risk detected (i.e. HQ and HI < 1) for the oral consumption of an aerial sample of lemon balm (BT-46 see Table 7) sourced from Spain at the theoretical exposure levels modelled.

#### *Santolina chamaecyparissus* (cotton lavender)

This is the first elemental assessment of *S. chamaecyparissus* flower to our knowledge. Element profiling data is limited, with one study by Zekri et al.^139^ who described this plant as a Pb-excluder and Cd-accumulator. Pb levels were higher in root samples compared to aerial samples. Lower Pb levels (48.01 µg kg^−1^) were observed in cotton lavender flowers (BT-47) compared to the majority of other samples tested in this study, which aligns with Zekri et al.^139^. Based on the theoretical exposure levels modelled in this study, there is no appreciable risk detected (i.e. HQ and HI < 1; see Table 7) for the oral consumption of cotton lavender flowers sourced from Spain.

#### *Mentha* × *piperita* (peppermint)

Herbal and fruit infusion ingredients (HFIs) are dried plants, or parts thereof, that are used alone or in combination to prepare an infusion or decoction with freshly boiled water^51^. Aerial peppermint parts are commonly used as HFIs, while the essential oil is extensively used in the food and cosmetic industries. A previous study^140^ demonstrated that the use of Cu-enriched compost altered the chemoprofile of peppermint oil as well influencing Cu, Cd, Cr, Ni, Pb and Zn content of the oil. This further emphasizes the influence of certain element concentrations on the potential therapeutic activity of the extracted oil. In this present study, Co, Cr and Li concentrations are in good accordance with Lozak et al.^68^. Similarly, Cd is within the lower range of previous reports^25,71,141^. Metal transitions rates in peppermint infusions were reported for Cu (24–48%) and Pb (7–9%)^25,51^. Several reports noted the low extraction efficiency of Cd in peppermint infusions^25,51,68^. In a previous study, Cu, Pb and Zn distribution in peppermint decreased accordingly: roots > leaves > stem > flower, and for Cd: roots > flowers > leaves > stem^110^. The elevated levels observed in this study (BT-49) when compared to previous findings, could be related to the fact that we analysed the comminuted whole aerial plant parts (leaves, stem, flowers)—not the isolated part(s). This emphasises the importance of specifying the plant part analysed, and not just the species, to ascertain variations between plant tissues and organs. There was no risk detected (i.e. HQ and HI < 1; see Table 7) for the oral consumption of peppermint (BT-49) sourced from Spain at the theoretical exposure levels modelled in this study.

#### *Peumus boldus* molina (boldo)

Boldo leaf presented with the highest levels of Cr (4534.43 µg kg^−1^) out of all fifty samples analysed in this present study—which is higher than levels observed previously^25,142^. Levels of Cd^143^ are in accordance with previous reports. Our findings for Pb (129.26 µg kg^−1^) are in line with Milani et al.^25^, but much lower for Co, Hg and V compared to Silva et al.^142^. Based on the risk assessments in this current study, no risk was detected (i.e. HQ and HI < 1; see Table 7) for the oral consumption of boldo leaves sourced from Spain at the theoretical exposure levels modelled.

### Recommendations to facilitate botanical safety assessments

Health risk is primarily associated with duration^19^, and the rates of ingestion^144^. A current, major data gap in Europe is the considerable lack of *intake (consumption, occurrence) survey data* for medicinal herbs and botanicals ingredients^145^, despite evidence of increasing popularity among consumers^146^. Consumer intake data directly influences actual exposure and corresponding risk assessment predictions. It is vitally important with regards to actual contributions and realistic exposure scenarios^51^. Risk assessment methodologies often account for lifelong daily use which may not be representative for herbal/botanical preparations^13^. Shorter-than-lifetime use are often more reflective of real-life scenarios, considering intermittent, non-consecutive usage of herbal- or plant-food supplements, herbal medicinal products (HMPs) or herbal beverages^13^. Intake patterns are variable, from a few days, to a few years, to daily consumption^146^, which makes interpretation challenging. Integrating measures of prospective intake in national dietary surveys, albeit complex, would provide crucial data for botanical safety assessments at European level^147^.

The inclusion of realistic exposure scenarios can also help contextualise analytical findings. Generic IR(D)’s of 200 and 500 g day^−1^, signifying the mean and 95th percentile (maximum daily dosage) of Chinese Herbal Medicinal Products, respectively, was proposed in the 2020 Chinese Pharmacopeia^15^ and validated in recent health risk assessments of herbal preparations^8,18^. These intake values are considerably higher than those [infrequently] quoted by European counterparts regarding medicinal herb consumption^13,51,148^. According to a National Food Consumption Survey (Germany), a herbal tea intake of 0.093 g (kg BW)^−1^d^−1^ was estimated, equating to an average 6.5 g day^−1^ (adult; 70 kg BW) for high consumers^51^, corresponding to the preparation of a water-based herbal tea/infusion (10 g plant material per 1L). Another study assumed realistic and worst-case scenario daily intakes (mL = g) of 95.4 mL and 363 mL (children; 39.7 kg BW), 194.7 mL and 1 L (adult female; 63.6 kg BW), 114 mL and 600 mL (adult male; 81.5 kg BW)^148^, again for herbal tea/infusions only. Recently, Chen et al.^13^ surrogated a lifetime exposure of 200 mg day^−1^ in their risk assessment of herbal products, modelled on data described by EFSA^149^. Considering the absence of a validated European equivalent, we therefore opted to implement 200 g day^−1^ to represent a theoretical maximum or “*conservative*” IR(D)^15^ and a theoretical minimum IR(D) of 200 mg day^−1^^13^ to represent a more “*realistic*” exposure scenario, in the risk assessment equations outlined earlier.

An additional consideration for the analysis of herbal preparations (e.g. teas, decoctions, tinctures) is *metal ion solubility* and the associated *metal ion transition rate* (%). Estimations involve the comparison of the metal concentration in the raw (fresh or dried) plant material to the final preparation at a specific volume as described earlier^51^, and prior referred to as the “*leaching efficiency*”^144^. Some authors suggest that the metal transition rates in herbal teas/infusions are influenced by the matrix (i.e. plant species), origin, grade (i.e. tea leaf grade), particle size, processing techniques and mode of preparation (i.e. infusion duration, water temperature)^51^. Milani et al.^25^ categorised Al, As, Ba, Sc, Cr, Fe, Pb and Se as poorly extractable and Cu, Mn, Ni and Zn, as moderately extractable in herbal infusions. Two hypothetical exposure scenarios proposed by Harris et al.^144^ were referred to as the “*most likely*” and the “*most conservative*”, referring to acute exposure with 10% leaching (i.e. from plant material to final product/ preparation), and chronic exposure at 100% leaching, respectively. Alternatively, a low, medium, and high (i.e. worst-case) theoretical transition rate of 10, 50 and 100% could be implemented in calculations to have a more representative suite of [metal-to-preparation] transition rates.

In the absence of either a standardised/theoretical universal transfer rate or an experimental transfer rate specific to each metal and plant matrix analysed, generic assumptions have been applied in studies however this may not be truly representative and could lead to over- or under-estimations. Transition rates are highly variable between analytes and samples—Zuo et al.^18^ reported that the average metal transfer rate for Chinese HMP’s is ≤ 10%; while Schulzki^51^ and Luo et al.^8^ reported transfer rates of 16–92.2% (Cu) and 13.1–50.0% (Al); and 14% (Cd, Cu and Pb), 35% (As) and 24% (Hg), respectively. Development of a universal default transfer (or bioavailability) rate for risk assessment would facilitate further inter-study comparisons. The transfer rates and other relevant mathematical input criteria applied in this study are outlined in Table 4.

The carcinogenic risk (CR) assessment allows for the estimation of the possibility of a population developing cancer following exposure to a carcinogen^16^. Some studies report the carcinogenic risk of Cd, Cr, Pb and Ni using the incremental lifetime cancer risk (ILCR) equation, which is a probabilistic assessment of carcinogenic risk involving the multiplication of the estimated CDI over a lifetime (e.g. 70 years) by the corresponding cancer slope factor (CSF) for the carcinogenic substance (i.e. Cd, Cr, Pb and Ni), as shown in Table 4^16,17^. Level of risk can be categorised based on the Delphi method from < 10^–6^ (extremely low risk) to > 10^–3^ (extremely high risk)^16^. CR assessment was however excluded from this current study due to the unavailability of a validated method and the generally unexplained variations in the equation used in many studies assessing the CR of carcinogenic metals in botanical or herbal products^8,16,18^. Additionally, if considering supplementation or treatment with PFS and/or HMPs, intermittent exposure scenarios may be more representative and thus guidance on the estimated frequency and duration (EF, ED) is necessary to ensure robust estimations. Considering that the IARC classifies Be, Cd, Cr(VI) and Ni as Group 1 compounds (*carcinogenic to humans*), Pb as Group 2A (*probable carcinogens*) and Co as Group 2B (*possible carcinogens*)^150–153^, the standardised assessment of the carcinogenic risk of these hazardous contaminants is essential in the context of public health.

Standardisation and/or official guidance on risk assessment input parameters and criteria would critically support future inter-study comparison in this area of research, and thus, help assure botanical safety.

## Conclusion

Knowledge of element concentrations in botanical material is relevant to many industries, and as the portfolio of plant-based products increases worldwide, monitoring of their quality and safety is critical to help assure consumer protection. The primary aim of this study was to quantify the levels of multiple elements (*n* = 22) in a diverse range of botanical samples (*n* = 50), and to estimate any potential health risks from oral exposure to potentially harmful elements. Based on our findings, consumption of the plants under investigation are considered safe and of no risk to consumers at lower intake rates (HQ and HI < 1). At higher intake levels, there is an increased health risk (HQ > 1) from Cd (arnica, dandelion, yarrow, borage, mugwort, marshmallow), and Pb (hawthorn, dandelion, comfrey, great mullein) exposures. A further 42% of samples were categorised as potentially unsafe (HI ≥ 1) regarding cumulative exposure to Cu, Cd, Hg and Pb, following high consumption of hawthorn, arnica, dandelion, marigold, nettle, yarrow, comfrey, borage, coltsfoot, birds-foot trefoil.

Key findings from the PCA revealed novel trends which suggest a potential influence from post-harvest processing methods on Cr, Ti and V levels in commercially-acquired plant material, in comparison to wild-collected and farm-grown plants. Furthermore, levels of Mo and Ni appeared higher in wild-collected plants, and a strong correlation was observed between Pb-Bi, Be-V, Bi-Sn, and Tl-Mo occurrence within all botanical samples.

From a regulatory and policy perspective, there is an explicit need for further data within the botanical sciences. This study provides a robust blueprint method and novel reference profile(s) for the evaluation of essential elements and/or metal contaminants in plants for use in quality investigations (e.g., authentication and adulteration), nutritional analysis and even phytoremediation studies. It is hoped that studies, such as the present investigation, can contribute data that will influence the future development of (inter)-national policies and/or guidance documentation for the harmonised management of botanical ingredients.

## Supplementary Information

Supplementary Table S1.

## Data Availability

All data generated or analysed during this study are included in this published article (and its Supplementary Information files).
