# Supplementary material for: Novel metallomic profiling and non-carcinogenic risk assessment of botanical ingredients for use in herbal, phytopharmaceutical and dietary products using HR-ICP-SFMS
Source: Sci Rep. 2022 Oct 20;12:17582. doi: 10.1038/s41598-022-16873-1 (PMC9584900; doi:10.1038/s41598-022-16873-1)
Supplement: Supplementary file 1 — Supplementary Table S1. [file 41598_2022_16873_MOESM1_ESM.pdf]

Supplementary Information

Novel metallomic profiling and non-carcinogenic risk assessment of botanical ingredients for use in herbal, phytopharmaceutical and dietary products using HR-ICP-SFMS.

Ciara-Ruth Kenny, Ph.D. [a,b] (ciararuth.kenny@mtu.ie), Gavin Ring [b] (gavin.ring@mycit.ie), Aisling Sheehan [b] (aisling.sheehan@mycit.ie), Michael A.P. Mc Auliffe, Ph.D. [c] (Michael.McAuliffe@mtu.ie), Brigid Lucey, Ph.D.[a] (Brigid.Lucey@mtu.ie), and Ambrose Furey, Ph.D. [a,b*] (ambrose.furey@mtu.ie)

[a] CREATE (Centre for Research in Advanced Therapeutic Engineering) and BioExplore, Department of Biological Sciences, Munster Technological University (MTU), Rossa Avenue, Bishopstown, Co. Cork, T12 P928, Ireland

[b] Department of Physical Sciences, Munster Technological University (MTU), Rossa Avenue, Bishopstown, Co. Cork, T12 P928, Ireland

[c] Centre for Advanced Photonics and Process Analysis, Munster Technological University (MTU), Rossa Avenue, Bishopstown, Co. Cork, T12 P928, Ireland.

*Corresponding author (Tel. +353-21-4335875)

Table S1: Correlation Matrix

	^{7}Li	^{9}Be	^{95}Mo	^{111}Cd	^{118}Sn	^{137}Ba	^{195}Pt	^{202}Hg	^{205}Tl	^{208}Pb	^{209}Bi	^{47}Ti	^{51}V	^{52}Cr	^{59}Co	^{60}Ni	^{63}Cu
^{7}Li	1	0.1940171	0.3748085	0.2278636	0.1783769	0.1144627	-0.03972002	0.2805984	0.2995773	0.3092919	0.2462267	0.01690004	0.0797407	-0.02006666	0.4022633	0.2501423	0.0889049
^{9}Be	0.1940171	1	-0.08774804	0.3724169	0.3613077	0.163011	0.2826686	0.2759373	0.05019184	0.5056304	0.4593908	0.5534157	0.7573687	0.5840721	0.4687898	-0.006479476	-0.06968638
^{95}Mo	0.3748085	-0.08774804	1	0.1017865	0.5124262	0.1240325	-0.04336336	0.115552	0.53553	0.1897952	0.4156547	-0.07706568	-0.04874843	-0.09486218	-0.1248636	0.1721659	0.2876109
^{111}Cd	0.2278636	0.3724169	0.1017865	1	0.223854	0.1962733	0.2511126	0.09219526	0.1196295	0.209473	0.2168606	0.2949241	0.2026486	0.3779152	0.3340683	0.3510485	-0.001666458
^{118}Sn	0.1783769	0.3613077	0.5124262	0.223854	1	0.1636281	0.3443955	0.3660917	0.3028796	0.3408298	0.6066924	0.1276024	0.2879663	0.1910929	0.08894809	-0.2063489	0.1517621
^{137}Ba	0.1144627	0.163011	0.1240325	0.1962733	0.1636281	1	0.1335462	0.2462643	0.1243898	0.1787577	0.1813965	0.2537692	0.2193193	0.1339978	0.1107424	0.1770369	-0.1304589
^{195}Pt	-0.03972002	0.2826686	-0.04336336	0.2511126	0.3443955	0.1335462	1	0.2420091	-0.07292377	-0.00685902	0.01368637	0.03059316	0.261041	0.3109444	0.2304173	0.006551383	0.06961475
^{202}Hg	0.2805984	0.2759373	0.115552	0.09219526	0.3660917	0.2462643	0.2420091	1	0.0606098	0.4987507	0.4626849	0.155453	0.1310906	0.2371006	0.2411557	-0.09958265	0.04632163
^{205}Tl	0.2995773	0.05019184	0.53553	0.1196295	0.3028796	0.1243898	-0.07292377	0.0606098	1	0.3157923	0.5117241	0.2069141	0.2623434	0.2061414	0.06099944	0.2500152	0.1554103
^{208}Pb	0.3092919	0.5056304	0.1897952	0.209473	0.3408298	0.1787577	-0.00685902	0.4987507	0.3157923	1	0.8434514	0.4328104	0.482073	0.3290921	0.3497063	0.0009386507	-0.005748771

^{209}Bi	0.2462267	0.4593908	0.4156547	0.2168606	0.6066924	0.1813965	0.01368637	0.4626849	0.5117241	0.8434514	1	0.2960882	0.4179654	0.2670815	0.2640849	-0.03462559	0.07546452
^{47}Ti	0.01690004	0.5534157	-0.07706568	0.2949241	0.1276024	0.2537692	0.03059316	0.155453	0.2069141	0.4328104	0.2960882	1	0.6879956	0.530987	0.3465901	0.03584969	-0.2557985
^{51}V	0.0797407	0.7573687	-0.04874843	0.2026486	0.2879663	0.2193193	0.261041	0.1310906	0.2623434	0.482073	0.4179654	0.6879956	1	0.5175784	0.4403328	0.1083418	-0.1130748
^{52}Cr	-0.02006666	0.5840721	-0.09486218	0.3779152	0.1910929	0.1339978	0.3109444	0.2371006	0.2061414	0.3290921	0.2670815	0.530987	0.5175784	1	0.2659758	0.2702496	0.1283098
^{59}Co	0.4022633	0.4687898	-0.1248636	0.3340683	0.08894809	0.1107424	0.2304173	0.2411557	0.06099944	0.3497063	0.2640849	0.3465901	0.4403328	0.2659758	1	0.4328619	-0.1766916
^{60}Ni	0.2501423	-0.006479476	0.1721659	0.3510485	-0.2063489	0.1770369	0.006551383	-0.09958265	0.2500152	0.0009386507	-0.03462559	0.03584969	0.1083418	0.2702496	0.4328619	1	0.1720193
^{63}Cu	0.0889049	-0.06968638	0.2876109	-0.001666458	0.1517621	-0.1304589	0.06961475	0.04632163	0.1554103	-0.005748771	0.07546452	-0.2557985	-0.1130748	0.1283098	-0.1766916	0.1720193	1

Element concentration: $\mu g.kg^{-1}$
